# Stem cell transplantation extends the reproductive life span of naturally aging cynomolgus monkeys

**DOI:** 10.1038/s41421-024-00726-4

**Published:** 2024-11-05

**Authors:** Long Yan, Wan Tu, Xuehan Zhao, Haifeng Wan, Jiaqi Wu, Yan Zhao, Jun Wu, Yingpu Sun, Lan Zhu, Yingying Qin, Linli Hu, Hua Yang, Qiong Ke, Wenzhe Zhang, Wei Luo, Zhenyu Xiao, Xueyu Chen, Qiqian Wu, Beijia He, Man Teng, Shanjun Dai, Jinglei Zhai, Hao Wu, Xiaokui Yang, Fan Guo, Hongmei Wang

**Affiliations:** 1 Key Laboratory of Organ Regeneration and Reconstruction, State Key Laboratory of Stem Cell and Reproductive Biology, Institute of Zoology, Chinese Academy of Sciences, Beijing, China; 2 University of Chinese Academy of Sciences, Beijing, China; 3 Institute for Stem Cell and Regeneration, Chinese Academy of Sciences, Beijing, China; 4 Beijing Institute for Stem Cell and Regenerative Medicine, Beijing, China; 5 Department of Human Reproductive Medicine, Beijing Obstetrics and Gynecology Hospital, Capital Medical University. Beijing Maternal and Child Health Care Hospital, Beijing, China; 6 National Stem Cell Resource Center, Chinese Academy of Sciences, Beijing, China; 7 Center for Reproductive Medicine, Henan Key Laboratory of Reproduction and Genetics, The First Affiliated Hospital of Zhengzhou University, Zhengzhou, Henan China; 8 Department of Obstetrics and Gynecology, National Clinical Research Center for Obstetric & Gynecologic Diseases, Peking Union Medical College Hospital, Chinese Academy of Medical Sciences & Peking Union Medical College, Beijing, China; 9 State Key Laboratory of Reproductive Medicine and Offspring Health, Center for Reproductive Medicine, Institute of Women, Children and Reproductive Health, Shandong University, Jinan, Shandong China; 10 The First Affiliated Hospital, Sun Yat-sen University, Guangzhou, Guangdong China; 11 Department of Obstetrics and Gynecology, Shandong Provincial Hospital, Affiliated to Shandong First Medical University, Jinan, Shandong China; 12 School of Life Science, Beijing Institute of Technology, Beijing, China; 13 Laboratory of Neonatology, Department of Neonatology, Affiliated Shenzhen Maternity & Child Healthcare Hospital, Southern Medical University, Shenzhen, Guangdong China

**Keywords:** Bioinformatics, Mesenchymal stem cells, Ageing

## Abstract

The ovary is crucial for female reproduction and health, as it generates oocytes and secretes sex hormones. Transplantation of mesenchymal stem cells (MSCs) has been shown to alleviate pathological ovarian aging. However, it is unclear whether MSCs could benefit the naturally aging ovary. In this study, we first examined the dynamics of ovarian reserve of Chinese women during perimenopause. Using a naturally aging cynomolgus monkey (*Macaca fascicularis*) model, we found that transplanting human embryonic stem cells-derived MSC-like cells, which we called M cells, into the aging ovaries significantly decreased ovarian fibrosis and DNA damage, enhanced secretion of sex hormones and improved fertility. Encouragingly, a healthy baby monkey was born after M-cell transplantation. Moreover, single-cell RNA sequencing analysis and in vitro functional validation suggested that apoptosis, oxidative damage, inflammation, and fibrosis were mitigated in granulosa cells and stromal cells following M-cell transplantation. Altogether, these findings demonstrate the beneficial effects of M-cell transplantation on aging ovaries and expand our understanding of the molecular mechanisms underlying ovarian aging and stem cell-based alleviation of this process.

## Introduction

The ovary is a critical female reproductive organ, serving as the source of oocytes and sex hormones. Ovarian function is highly dependent on the ovarian reserve, which refers to the number of primordial follicles housed in the ovary cortex^1^. The follicle is the functional unit of the ovary and is comprised of an oocyte surrounded by granulosa cells (GCs) and theca cells (TCs)^2^. Women are born with a finite stock of primordial follicles, forming their initial ovarian reserve^3^. After women enter puberty, a large number of primordial follicles are regularly recruited and developed into growing follicles. As a result, the ovarian reserve gradually diminishes with aging^1,4^, eventually resulting in menopause^5^. The ovarian reserve, especially during the perimenopausal period, is of great importance and has been extensively studied in European and North American populations. It has been reported that a small number of primordial follicles were still retained in the perimenopausal ovary^6,7^. This is extremely exciting, as making these primordial follicles functional could potentially extend women’s reproductive life span and delay the onset of menopause. The age at the onset of menopause is variable across different demographics^8^, illustrating women from different races may have different sizes of ovarian reserve and rates of ovarian reserve decline. Unfortunately, no research on the ovarian reserve of Asian women has been reported. Chinese women experience notable fertility decline at ~37 years old (y)^9^, enter perimenopause at 46 y^10^, and typically reach menopause at ~48.6 y^11,12^. It is pivotal to explore the dynamic ovarian reserve of Chinese women during the perimenopausal period.

Menopause is characterized by reduced secretion of estradiol (E2) and progesterone (P4), amenorrhea, as well as ovarian and endometrial atrophy. Women at menopause may suffer from osteoporosis^13^, cardiovascular^14^, and neurodegenerative diseases^15^. More significantly, the average life expectancy in women has climbed over the past few decades, while the menopausal age has barely changed^8^, indicating that women are now spending more than one-third of their lives after menopause^16^. Therefore, alleviating ovarian aging is critical for women’s long-term health. Hormone replacement therapy (HRT) is usually recommended to alleviate menopausal symptoms as a result of estrogen deficiency^17^. However, there is little evidence regarding the optimal type, regimen, and dosing of HRT^18^. Moreover, HRT increases the risks of chronic diseases such as coronary heart disease, invasive breast cancer, and stroke^19^. It is pivotal to develop more secure and more effective strategies to alleviate natural ovarian aging based on a precise understanding of the nature of ovarian aging.

Ovarian aging is characterized by high levels of oxidative stress (OS), DNA damage, inflammation, and fibrosis^20–22^. Ovarian aging has been demonstrated to be highly correlated with reactive oxygen species (ROS)^23^. Excessive ROS produced by the mitochondrial respiratory chain leads to an imbalance of redox reactions and induces an OS state in the body, while OS has been proven to be pivotal in the pathogenesis of age-related infertility in females^24^. A large amount of ROS causes DNA damage via modifying nucleotides, breaking DNA strands, and introducing mutations^25^. DNA damage can lead to chromosomal abnormalities that potentially lead to infertility and ovarian aging^26^. Moreover, ROS accumulation initiates a pro-inflammatory effect by activating the nuclear transcription factor-kappaB^27,28^. It was reported that activation of inflammatory pathways in the ovary might disrupt follicular development and decrease oocyte quality^29^. Inflammation is a crucial trigger for fibrosis^30^, which causes impaired ovulation and ovarian wound healing^31,32^. It is pivotal to develop novel therapies that fundamentally activate the ovary by reducing oxidative damage, cell senescence, inflammation, and fibrosis, thus promoting follicle development and restoring ovarian function.

Mesenchymal stem cell (MSC)-based therapy has successfully recovered fertility in animal models or women with premature ovarian insufficiency^33–37^, suggesting the potential of this strategy in ameliorating physiological ovarian aging. Injection of MSCs derived from juvenile macaques into aged macaques via the femoral vein has been reported to reverse ovarian aging of the elderly individuals^38^. However, the therapeutic mechanisms underlying MSC treatment are far from clearly demonstrated. Furthermore, the approach to obtaining MSCs from young individuals restricts its potential application in clinical settings. Recently, a new type of stem cells, namely human embryonic stem cells (hESC)-derived MSC-like cells (abbreviated as M cells), which resemble MSCs, has gained attention as it possesses stronger immunomodulatory and anti-fibrotic functions and can be manufactured on a large scale^39^. Therefore, it is worthwhile to study the safety and efficacy of M-cell therapy in ameliorating physiological ovarian aging in animal models very close to humans, such as non-human primates.

In this study, we first determined the ovarian reserve of perimenopausal women in China through a quantitative morphometric study. Secondly, we transplanted M cells into the ovaries of naturally aging cynomolgus monkeys using laparoscopy and found that their ovarian function was restored, with one of the treated monkeys conceiving a healthy baby. Then, we performed single-cell RNA sequencing (scRNA-seq) on the ovarian tissues at different intervals after treatment to explore the alterations at the transcriptional level. We further validated the effects of M cells by co-culturing them with the senescent KGN cells and explored the roles of *PPARG*, *PRDX4*, and *RDX* during GC aging. Taken together, our data proved that the ovarian function of naturally aging cynomolgus monkeys could, in principle, be restored by M-cell transplantation and elucidated the underlying molecular mechanisms, suggesting that M-cell transplantation is a feasible clinical strategy to alleviate physiological ovarian aging and extend fertility life span.

## Results

### Deciphering the ovarian reserve of Chinese women at perimenopause

**Fig. 1:**
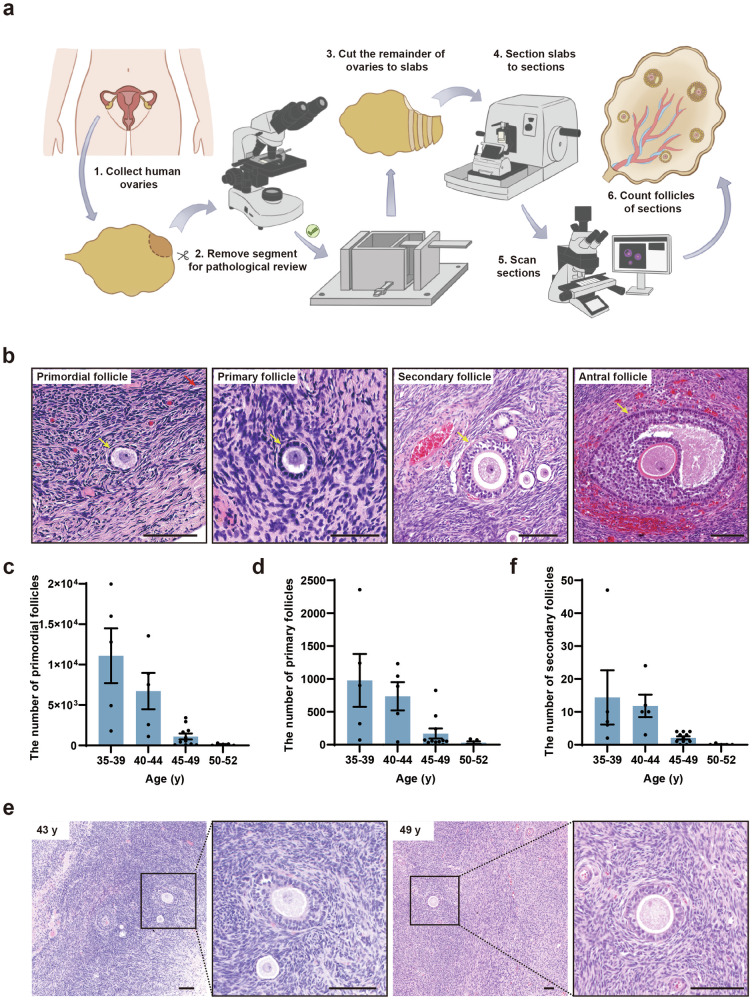
The ovarian reserve in perimenopausal Chinese women. **a** Schematic illustration of the experimental design for follicle counting. **b** H&E-stained ovarian sections of a 36-year-old (y) woman showed the normal morphology of primordial, primary, secondary, and antral follicles (yellow arrows). Scale bars, 100 μm. **c**, **d** Bar graphs illustrated the number of primordial (**c**) and primary (**d**) follicles in the 35–52 y women’s ovaries. *n* = 5 (35–39 y), *n* = 5 (40–44 y), *n* = 11 (45–49 y), *n* = 5 (50–52 y). **e** H&E-stained ovarian sections of 43 y and 49 y women showed the morphology of secondary follicles. Scale bars, 100 μm. **f** Bar graph illustrated the number of secondary follicles in the ovaries of 35–52 y. *n* = 5 (35–39 y), *n* = 5 (40–44 y), *n* = 11 (45–49 y), *n* = 5 (50–52 y).

To determine the ovarian reserve of Chinese women at perimenopause, we collected 28 ovaries from 26 Chinese women aged 35–52 y (Supplementary Fig. S1a and Table S1) and categorized them into four groups at five-year intervals: 35–39 y, 40–44 y, 45–49 y, and 50–52 y. We fixed the ovaries and cut them into 1-mm slabs perpendicular to the long axis of the ovary (Fig. 1a). Approximately one-third of the slabs were then selected following systematic random sampling rules^40^ and each slab was cut into 5-μm-thick sections. Next, five serial sections from every 50 sections were collected and stained with hematoxylin and eosin (H&E) and subjected to scanning for whole-slide images (Supplementary Fig. S1b). Subsequently, the images were subjected to follicle counting and ovarian reserve estimation using the method we previously developed^1^. Follicles at different stages were identified in the ovarian sections by H&E (Fig. 1b). We counted the primordial follicles characterized by a layer of flattened GCs embracing the oocyte (Fig. 1b). Follicles were counted only when a clearly defined oocyte nucleolus was observed. As the follicle counts between the two ovaries of the same woman were similar, we averaged the number of follicles of bilateral ovaries. As shown in Fig. 1c, Supplementary Tables S2, S3, the average number of primordial follicles per ovary decreased from 11,098 during 35–39 y to 6728 during 40–44 y. Then the number was drastically reduced to 1019 at 45–49 y. During the age of 50–52, 151 primordial follicles remained in the ovary. Furthermore, to elucidate whether the size of oocytes within primordial follicles changes with age, which may influence the oocyte quality, we calculated the average diameter of oocytes and oocyte nuclei of primordial follicles (Supplementary Fig. S1c). We found that the diameters of oocyte and oocyte nuclei of primordial follicles were comparable between young and aging ovaries, which indicated that the size of the primordial follicle did not change with age (Supplementary Fig. S1c). To determine whether folliculogenesis still occurs in perimenopausal ovaries, we examined the number of primary follicles. The number of primary follicles decreased with age: 978 on average at 35–39 y, 736 on average at 40–44 y, 161 on average at 45–49 y, and 39 on average at 50–52 y (Fig. 1d). The ratio of primary to primordial follicles increased with age, suggesting a rapid decline in the number of primordial follicles (Supplementary Fig. S1d). In addition, some primary follicles could further develop into secondary follicles in the 49 y-ovary (Fig. 1e, f). These results suggested that the primordial follicles in the ovaries of perimenopausal women can still develop into growing follicles.

To examine whether the dynamics of ovarian reserve are accompanied by microenvironmental changes in human ovaries, we measured the levels of fibrosis in perimenopausal ovaries. The Masson’s trichrome staining was performed to evaluate ovarian fibrosis. We found that fibrosis increased remarkably from 37 y to 50 y, and ovarian fibrotic levels at these stages were significantly higher than that at 23 y (Supplementary Fig. S1e, f). Taken together, we revealed the existence of primordial follicles in the ovaries of perimenopausal Chinese women and that the decline of ovarian reserve was accompanied by extended ovarian fibrosis.

### Xenotransplantation of M cells restored the ovarian function of naturally aging cynomolgus monkeys

**Fig. 2:**
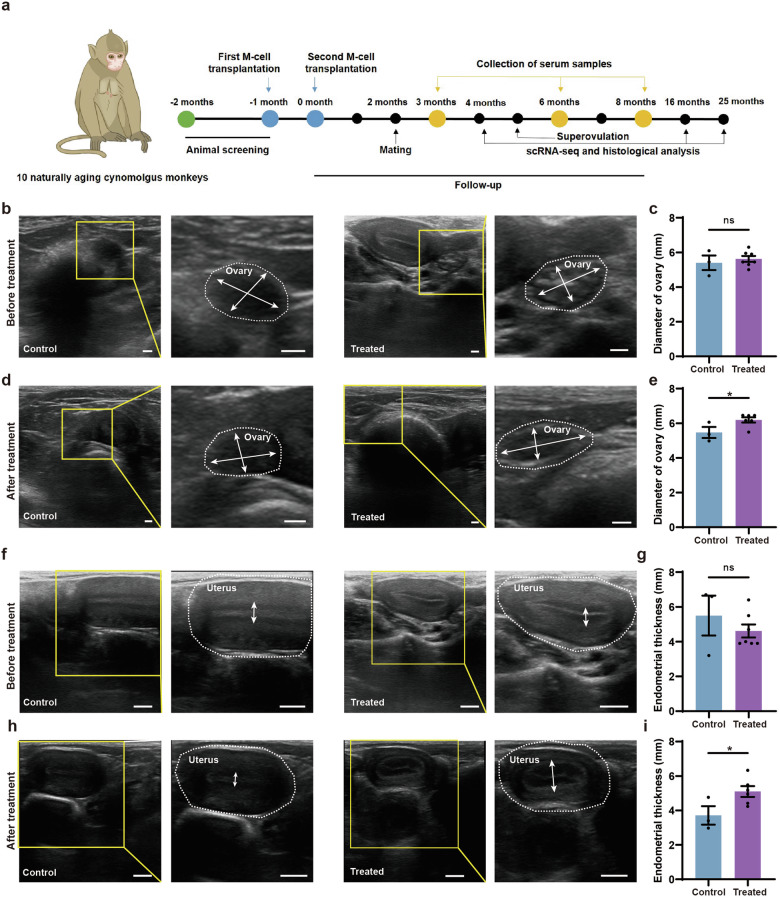
The morphological alterations of ovaries and uteruses in naturally aging monkeys after M-cell transplantation. **a** Experimental design for naturally aging cynomolgus monkeys in the control and treated group. **b**, **c** Ultrasound observation of the ovaries (**b**) and statistics of the ovarian diameters (**c**) before treatment. The yellow frames highlighted the regions zoomed in. White broken circles represented the ovaries. White arrows referred to the maximal widths and maximal lengths of the ovaries. Scale bars, 2 mm. *n* = 3 monkeys (control), *n* = 7 monkeys (treated). ns no significance (two-tailed *t*-test). **d**, **e** Ultrasound observation of the ovaries (1 month after treatment) (**d**) as well as statistics of ovarian diameters in control and treated groups. The final value for each monkey is the average of six measurements taken at 1, 2, 3, 4, 5, and 6 months of treatment (**e**). The yellow frames highlighted the regions zoomed in. White broken circles represented the ovaries. White arrows referred to the maximal widths and maximal lengths of the ovaries. Scale bars, 2 mm. *n* = 3 monkeys (control), *n* = 6 monkeys (treated, except the pregnant monkey T02), *P < 0.05 (two-tailed *t*-test). **f**, **g** Ultrasound observation of the uteri (**f**) and statistics of the endometrial thicknesses of naturally aging monkeys (**g**) before treatment. The yellow frames highlighted the regions zoomed in. White broken circles represented the uteri. White arrows referred to the endometrial thicknesses. Scale bars, 5 mm. *n* = 3 monkeys (control), *n* = 7 monkeys (treated). ns, no significance (two-tailed *t*-test). **h**, **i** Ultrasound observation of the uteri (4 months after treatment) (**h**) as well as statistics of endometrial thicknesses in control and treated groups. The final value for each monkey is the average of six measurements taken at 1, 2, 3, 4, 5, and 6 months of treatment (**i**). The yellow frames highlighted the regions zoomed in. White broken circles represented the uteri. White arrows referred to the endometrial thicknesses. Scale bars, 5 mm. *n* = 3 monkeys (control), *n* = 6 monkeys (treated, except the pregnant monkey T02). *P < 0.05 (two-tailed *t*-test).

The similarities between human and monkey ovarian aging make the monkey an excellent model to study the safety and efficacy of M-cell transplantation in ameliorating human physiological ovarian aging^1,41^. The menopausal transition is marked by increased variability in menstrual cycle length and reproductive endocrine abnormalities. We selected perimenopausal cynomolgus monkeys based on their age, menstrual cycle, morphology of the ovary and uterus under ultrasound, as well as sex hormone level (Fig. 2a). As species of the genus *Macaca* undergo menopause at ~25 y^42^, we selected 10 cynomolgus monkeys aged 18–23 y. All the monkeys manifested menstrual irregularities, suggesting that they entered perimenopause (Supplementary Table S4). Ultrasound observation showed that the average diameter of ovaries and thickness of endometria of these monkeys were 5.56 ± 0.16 mm and 4.88 ± 0.41 mm, respectively. The average E2 at the early follicular phase of the menstrual cycle was 56.63 ± 6.2 pg/mL, which was lower than that of young monkeys reported in previous studies^43–45^. These results indicated that the selected monkeys were experiencing perimenopause and were suitable for the study of M-cell therapy on alleviating natural ovarian aging.

**Fig. 3:**
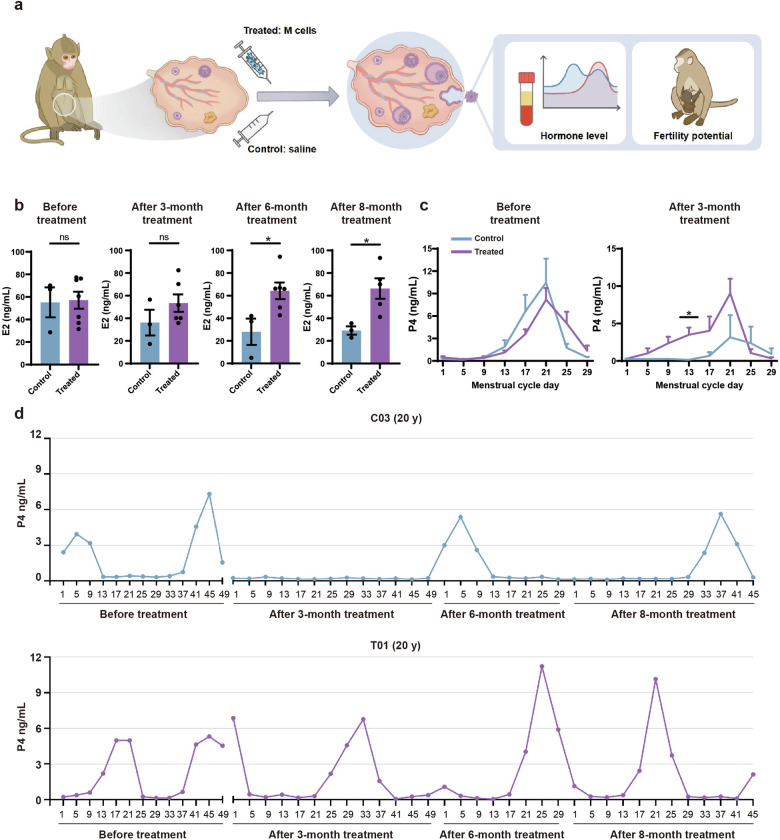
Transplantation of M cells increased E2 and P4 levels of the naturally aging monkeys. **a** Experimental design to test the effects of M-cell transplantation on hormone level and fertility potential of naturally aging monkeys. **b** Bar graphs illustrated the levels of E2 at the early follicular phase of the menstrual cycle in control and treated monkeys before treatment and after 3-, 6-, 8-month treatment. Before treatment, *n* = 3 monkeys (control), *n* = 7 monkeys (treated). After 3- and 6-month treatment, *n* = 3 monkeys (control), *n* = 6 monkeys (treated, except the pregnant monkey T02). After 8-month treatment, *n* = 3 monkeys (control), *n* = 5 monkeys (treated, except the monkey T02 due to lactation period and T07 due to poor healthy condition during this period), ns no significance, *P < 0.05 (two-tailed *t*-test). **c** Line graphs illustrated the P4 level of monkeys before (left panel) treatment and 3 months (right panel) after treatment. Before treatment, *n* = 3 monkeys (control), *n* = 7 monkeys (treated). After 3-month treatment, *n* = 3 monkeys (control), *n* = 6 monkeys (treated, except the pregnant monkey T02). *P < 0.05 (multiple *t*-tests). **d** Line plots illustrated the P4 levels of representative control (C03) (upper panel) and treated (T01) (lower panel) monkeys before treatment and after 3-, 6- and 8-month treatment.

We randomly divided the monkeys into treated (*n* = 7) and control (*n* = 3) groups. The ages, ovarian diameters, and endometrial thicknesses, as well as E2 and P4 levels, were similar between the control and the treated groups (Figs. 2b, c, f, g, 3b, c). We administered two injections of M cells into the bilateral ovaries of the monkeys, spaced one month apart (Fig. 2a). M cells were derived from hESCs which were developed in our previous studies^39^. M cell-specific markers were confirmed to be expressed on the cells we derived (Supplementary Fig. S2). The treated group (T01–T07) was injected with 5 × 10^6^ M cells in 100 μL 0.9% saline per ovary while the control group (C01–C03) was injected with 100 μL 0.9% saline (Supplementary Fig. S3a). We assessed the safety and efficacy of M-cell transplantation over an 8-month follow-up. During this period, we measured body weights, traced menstruation, examined ovarian and uterine morphology via ultrasound scan, and detected sex hormone levels in the monkeys (Fig. 2a; Supplementary Fig. S3b). The cynomolgus monkeys were found healthy and none of them suffered from acute inflammation or malignant diseases until the last follow-up, which indicated the safety of M-cell transplantation. We also tested the fertility of these cynomolgus monkeys by superovulation or mating. Finally, we investigated transcriptomic alterations of monkey ovaries between control and treated groups by scRNA-seq and in vitro functional validation.

To examine the morphological changes of the ovary and uterus, we performed ultrasound scans of the control and treated monkeys after 1-, 2-, 3-, 4-, 5-, and 6-month treatment. The ovarian diameters of the treated group were significantly larger than those of the control group (Fig. 2d, e). The endometria of treated monkeys were notably thicker (5.07 ± 0.33 mm) than those of the control group (3.71 ± 0.54 mm) (Fig. 2h, i). These results suggested that M-cell transplantation may benefit follicle development and increase the endometrial thickness. To investigate whether M-cell transplantation could alter the levels of sex hormones in naturally aging cynomolgus monkeys, we collected the blood samples of aged monkeys from control and treated groups and measured E2 and P4 levels by an automated biochemical analyzer (Fig. 3a–c). The E2 levels were similar between control and treated groups after 3-month treatment (Fig. 3b). However, after 6-month (64.29 ± 17.94 pg/mL vs 28.07 ± 11.62 pg/mL) and 8-month (66.33 ± 9.03 pg/mL vs 29.22 ± 3.64 pg/mL) treatment, the serum E2 levels were significantly higher in the treated group compared with the control group (Fig. 3b; Supplementary Fig. S4). These results indicated that M-cell transplantation increased serum E2 levels and could persist till at least 8 months after treatment. The P4 levels were significantly higher in the treated group than those in the control group (Fig. 3c). Specifically, the serum P4 remained at a high level in most of the monkeys in the treated group after 3-month treatment. By comparison, the P4 levels of the control group reduced from 0 months to 8 months after saline injection (Fig. 3d; Supplementary Fig. S5). In summary, these results suggested that M-cell transplantation could effectively promote the sex hormone secretion of naturally aging cynomolgus monkeys.

### M-cell transplantation extended the reproductive life span of naturally aging cynomolgus monkeys

**Fig. 4:**
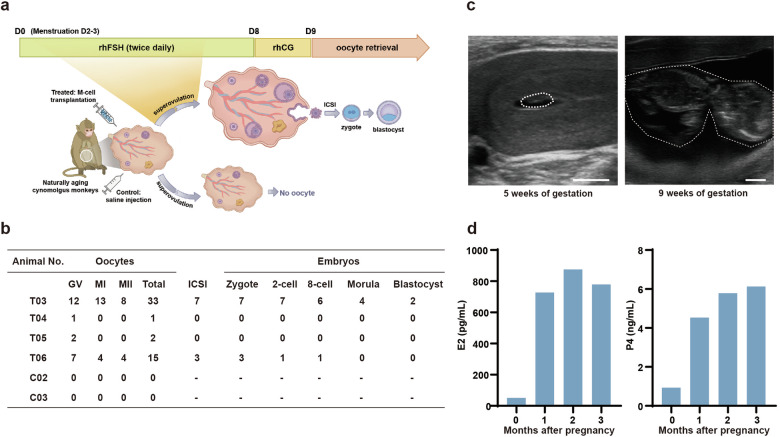
M-cell transplantation improved fertility of naturally aging monkeys. **a** Schematic diagram showed the experimental design of superovulation. After 5-month treatment, the monkeys were subjected to superovulation and ICSI experiments. **b** The numbers of oocytes obtained from the indicated monkeys by superovulation and the number of embryos developed to the indicated stages after ICSI. GV germinal vesicle. MI metaphase I, MII metaphase II. **c** Ultrasonographic of T02 at 5 and 9 weeks of gestation. The white broken circle represented the fetal capsule (left) or fetus (right). Scale bars, 5 mm. **d** Bar graphs illustrated serum levels of E2 and P4 before pregnancy and after 1-, 2-, and 3 months of pregnancy.

To determine whether M-cell transplantation improved the reproductive capacity of aging monkeys, we injected recombinant human follitropin-alpha (rhFSH) and recombinant human chorionic gonadotropin alpha (rhCG) intramuscularly to synchronously stimulate follicle growth of the monkeys after 5-month treatment (Fig. 4a). Monkeys C02 and C03 from the control group did not yield any oocytes, whereas T03–T06 in the treated group yielded 33, 1, 2, and 15 oocytes, respectively (Fig. 4b; Supplementary Fig. S6a). Two treated monkeys T03 and T06 yielded 8 and 4 metaphase II (MII) oocytes, respectively. To assess the fertilization capacity of these oocytes, 7 (T03) and 3 (T06) MII oocytes were collected for intracytoplasmic sperm injection (ICSI). All the mature oocytes subjected to ICSI could be successfully fertilized and 2 fertilized eggs from T03 successfully developed to the blastocyst stage (Fig. 4b; Supplementary Fig. S6a).

To verify whether the monkeys could achieve natural conception after M-cell transplantation, we transferred all the monkeys from control and treated groups into breeding cages two months after treatment and coupled them with fertile adult males for two months for natural mating (Supplementary Fig. S6b). All of these monkeys were then returned to single cages and continued to be monitored with regular follow-up tests. Encouragingly, one of the aging monkeys from the treated group got pregnant, whereas none of the control monkeys did (Fig. 4c). We observed the fetal capsule at 5 weeks of gestation and the fetus at 9 weeks of gestation by ultrasound scan, which indicated normal fetal development (Fig. 4c). In addition, we measured the sex hormone levels of the pregnant monkey and found that the serum level of E2 increased from 51.33 pg/mL before pregnancy to 779.4 pg/mL after 3-month pregnancy, and the P4 level increased from 0.94 ng/mL before pregnancy to 6.13 ng/mL after 3-month pregnancy (Fig. 4d). The pregnant monkey finally delivered a healthy full-term baby. The baby has grown up to 3 y and is healthy till now. The nutrition condition and the developmental status of this baby monkey (P1) are comparable to those of the offspring (5 males and 3 females of the same ages) delivered by young mothers (Supplementary Fig. S7 and Table S7). The preliminary data suggested that M-cell transplantation might improve the fertility potential of naturally aging cynomolgus monkeys.

**Fig. 5:**
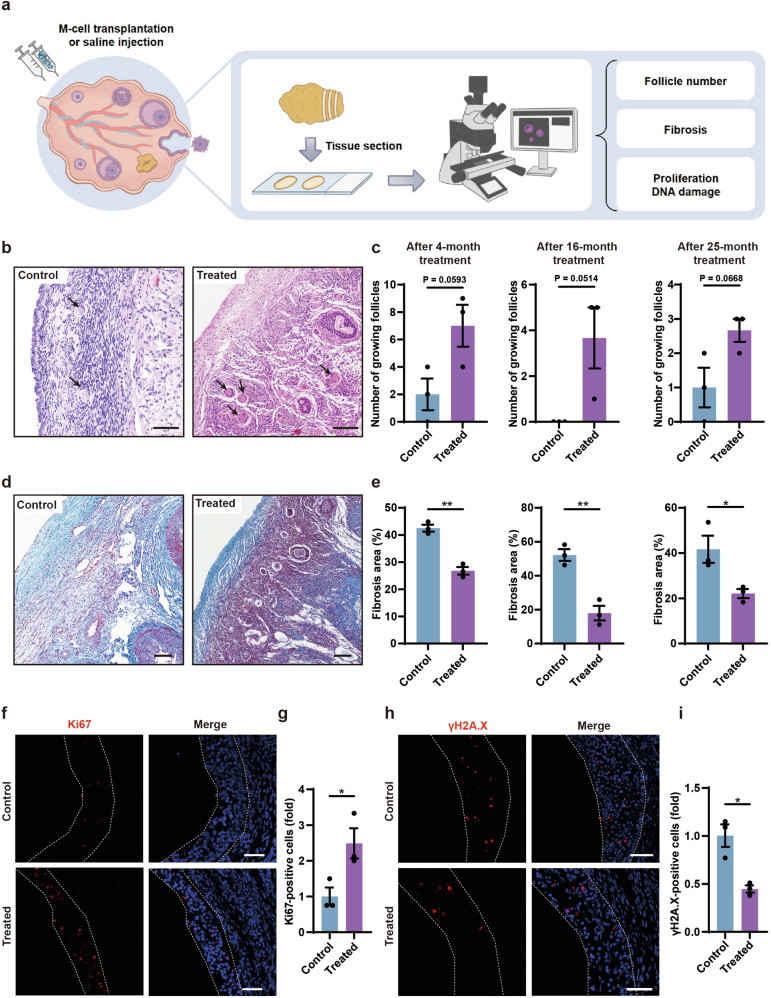
M-cell transplantation alleviated ovarian aging. **a** Experimental design to test the effects of M-cell transplantation on ovarian aging by histology. **b** H&E-stained ovarian sections showed growing follicles (black arrows) in the control and treated ovaries after 4-month treatment. Scale bars, 100 μm. **c** Bar graphs illustrated the numbers of growing follicles in the ovarian sections after 4-, 16-, and 25-month treatment. *n* = 3 sections, two-tailed *t*-test. **d** Masson’s trichrome staining showed fibrosis in the ovarian sections after 4-month treatment. Blue areas denoted collagen fibers (fibrosis). Red areas denoted muscle fibers and cytoplasm. Scale bars, 100 μm. **e** Bar graphs illustrated the proportion of fibrosis after 4- (left panel), 16- (middle panel), and 25- (right panel) month treatment. *n* = 3 sections, *P < 0.05, **P < 0.01 (two-tailed *t*-test). **f** Immunofluorescence analysis showed the expression of Ki67 in GCs of the treated group and the control group after 4-month treatment. White broken lines showed boundaries of GCs in antral follicles. Scale bars, 50 μm. **g** Bar graph showed the quantification of Ki67-positive GCs after 4-month treatment. *n* = 3 sections, *P < 0.05, two-tailed *t*-test. **h** Immunofluorescence analysis showed the expression of γH2A.X in GCs of the treated group and the control group after 4-month treatment. White broken lines showed boundaries of GCs in antral follicles. Scale bars, 50 μm. **i** Bar graph showed the quantificat**i**on of γH2A.X-positive GCs after 4-month treatment. *n* = 3 sections, *P < 0.05 (two-tailed *t*-test).

To further validate whether M cells could promote follicle development, we stained the ovarian sections of control and treated groups with H&E after 4-, 16-, and 25-month treatment and counted the number of growing follicles including primary follicles, secondary follicles, and antral follicles (Fig. 5a–c; Supplementary Table S5). The results showed a higher number of growing follicles in the treated group than in the control group (Fig. 5b, c; Supplementary Fig. S8). These results suggested that M-cell transplantation improved the fertility potential of naturally aging monkeys. To investigate whether M-cell transplantation could alleviate ovarian aging, we examined the aging properties in the ovaries of control and M cell-treated monkeys. To test whether the fibrosis in the monkey ovaries decreased after M-cell transplantation, we performed Masson’s trichrome staining on the ovarian sections from the control and treated monkeys after 4-, 16-, and 25-month treatment (Fig. 5d, e). Fibrosis was significantly decreased in the ovaries of the treated group than in the control group (Fig. 5e). GC proliferation is essential for the development of follicles which can be indicated by Ki67 staining^2^. To detect whether the proliferation of GCs in the ovaries increased after M-cell treatment, we performed immunofluorescent staining of Ki67 and found fewer proliferative cells in the control group, whereas more Ki67-positive GCs in the treated group after 4-month M-cell transplantation (Fig. 5f, g). To examine whether the DNA damage decreased after M-cell transplantation, we performed γH2A.X staining which represents DNA damage^21^. We found that γH2A.X-positive GCs in the ovaries of the treatment group were significantly reduced compared with those of the control group (Fig. 5h, i), which indicated that M cells attenuated ovarian DNA damage. Taken together, these results suggested that M-cell transplantation improved fertility, promoted follicular development and cell proliferation, as well as inhibited fibrosis and DNA damage.

### scRNA-seq analysis revealed potential molecular mechanisms whereby M-cell transplantation recovered ovarian function

**Fig. 6:**
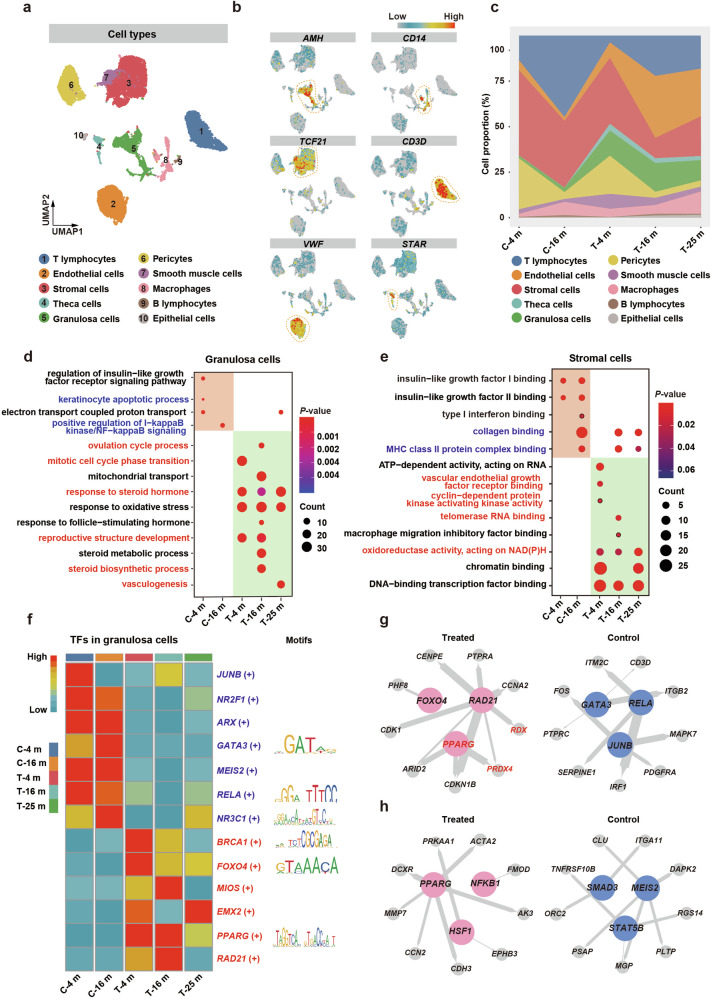
ScRNA-seq analysis unveiled potential regulatory networks and mechanisms underlying ovarian function restoration after M-cell transplantation. **a** Uniform manifold approximation and projection embedding visualization for 26,862 single cells from cynomolgus monkey ovarian tissues. **b** Visualized expression patterns of different cell type-specific genes. The color bar from blue to red indicated the relative expression levels from low to high. **c** Stacked area plot showed the fraction of major ovarian cell populations in all cynomolgus monkeys. Control group: after 4-month (C-4 m) and 16-month (C-16 m) saline injection; Treated group: after 4-month (T-4 m), 16-month (T-16 m), and 25-month (T-25 m) M-cell transplantation. **d** GO enrichment analysis of DEGs in granulosa cells of all groups. The color bar from blue to red indicated the *P*-value from low to high. Dot sizes indicated the gene counts in the corresponding GO item. **e** GO enrichment analysis of DEGs in stromal cells of all groups. The color bar from blue to red indicated the *P*-value from low to high. Dot sizes indicated the gene counts in the corresponding GO item. **f** Left panel: Heatmap showed regulon activity scores of the representative transcriptional regulons in granulosa cells of control and treated groups. Regulon activity scores were calculated by pySCENIC and were scaled for visualization. The color bar indicated the strength of regulon activity, with “High” (red) indicating active regulons and “Low” (blue) indicating inactive regulons. Right panel: Display of binding motifs of certain transcription factors. **g**, **h** The representative regulatory network in GCs (**g**) and SCs (**h**) of control and treated groups was identified using pySCENIC. Key transcriptional regulons were denoted as large colored circles, and corresponding target genes were denoted as small colored circles. The node size indicated the number of target genes, and the line thickness indicated the weight of the target regulon. The genes marked in red are implicated in alleviating ovarian aging.

To unravel the mechanisms underlying the recovery of ovarian function after M-cell transplantation in naturally aging cynomolgus monkeys, we collected ovaries from two control (4 and 16 months after treatment) and three M cell-treated monkeys (4, 16, and 25 months after treatment) and digested them into single-cell suspensions, which were subjected to scRNA-seq using the 10x Chromium system. After quality control, high-quality transcriptomes of 26,862 single cells were retained for further analyses (Supplementary Fig. S9a). Overall, we identified 10 major cell populations based on transcriptome profiles and signature genes of individual cells, containing all expected cell types except oocytes due to their scarcity in aging ovaries and the restriction of cell size in 10x Chromium system (Fig. 6a, b; Supplementary Fig. S9b). The proportions of GCs and TCs increased significantly after M-cell transplantation, with TCs appearing almost exclusively in the treated group (Fig. 6c). The percentage of endothelial cells and pericytes also increased markedly after M-cell transplantation, suggesting angiogenesis is more active in the treated group. Thus, these results indicated the recovery of follicular development after M-cell transplantation. Notably, the stromal cells (SCs) gradually decreased after M-cell transplantation, implying the alleviation of ovarian aging characteristics. Furthermore, the decreasing proportion of macrophages in the ovaries from the treated group after 4-month and 16-month treatment indicated that M cells reduced the inflammation of aging ovaries (Fig. 6c).

As GCs and SCs are essential for follicular development^22,46^, we took a closer look at them. First, we analyzed the gene expression patterns of GCs and SCs. Then, we identified differentially expressed genes (DEGs) and then conducted Gene Ontology (GO) analysis to predict what biological functions they may be involved in. Compared with GCs in the control group, upregulated genes in the GCs of the treated group were enriched for ovulation, response to the hormone, and cell proliferation, such as the “ovulation cycle process”, “response to steroid hormone”, and “mitotic cell cycle phase transition”. Downregulated genes in the GCs were enriched for apoptosis and inflammation, such as “keratinocyte apoptotic process” and “positive regulation of I-kappaB kinase/NF-kappaB signaling” (Fig. 6d). In SCs of the treated group, the upregulated genes were enriched for angiogenesis and proliferation, while the downregulated genes were enriched for fibrosis (Fig. 6e).

To identify the transcription factors (TFs) that may play important roles in M cell-induced benefits, we performed single-cell regulatory network inference and clustering (SCENIC) analysis to score the activity of gene regulatory networks (Fig. 6f; Supplementary Fig. S9c). To refine the aging-associated TFs in the context of M-cell transplantation, we compared the TFs identified in this study with the aging/longevity-associated genes annotated in the GenAge database and constructed the transcriptional regulatory networks of core transcriptional regulators and their target genes. We found that in GCs, *PPARG*, *RAD21, FOXO4*, and the target genes *PRDX4*, and *RDX*, which were essential for cell proliferation and response to estradiol, showed significant expression changes in the treated group, while *JUNB*, *RELA*, and *GATA3*, which were crucial for immune response activation, played master roles in the control group (Fig. 6g; Supplementary Fig. S9d). In SCs, *PPARG*, *NFKB1*, and *HSF1* involved in wound healing and response to oxygen levels were the master active TFs in the treated group, while *MEIS2, SMAD3*, and *STAT5B* involved in mitochondrial depolarization were the master active TFs in the control group (Fig. 6h; Supplementary Fig. S9e). Cell bidirectional communication via signal transduction is required for proper follicular development^46^. To investigate the cell–cell interaction between GCs and SCs, we conducted intercellular interaction analyses based on ligand–receptor pairs. We found that the COL1A1–integrin α2β1 pair related to collagen metabolism^47^ and VEGFB–FLT1 pair correlated with angiogenesis^48^ were enriched in the treated group (Supplementary Fig. S9f). Thus, the results illustrated that M-cell transplantation reduced fibrosis and enhanced angiogenesis of the aging ovaries, which further indicated better follicular development.

Taken together, the scRNA-seq analysis showed that M-cell transplantation reversed ovarian aging by promoting follicle development (increase of cell proliferation, angiogenesis, and hormone response), and decreasing inflammation, fibrosis, oxidative damage, and apoptosis.

### Cell-based assays validated the anti-inflammatory, anti-oxidative, and proliferation-promoting effects through the upregulation of *PPARG*, *PRDX4*, and *RDX* following M-cell transplantation

**Fig. 7:**
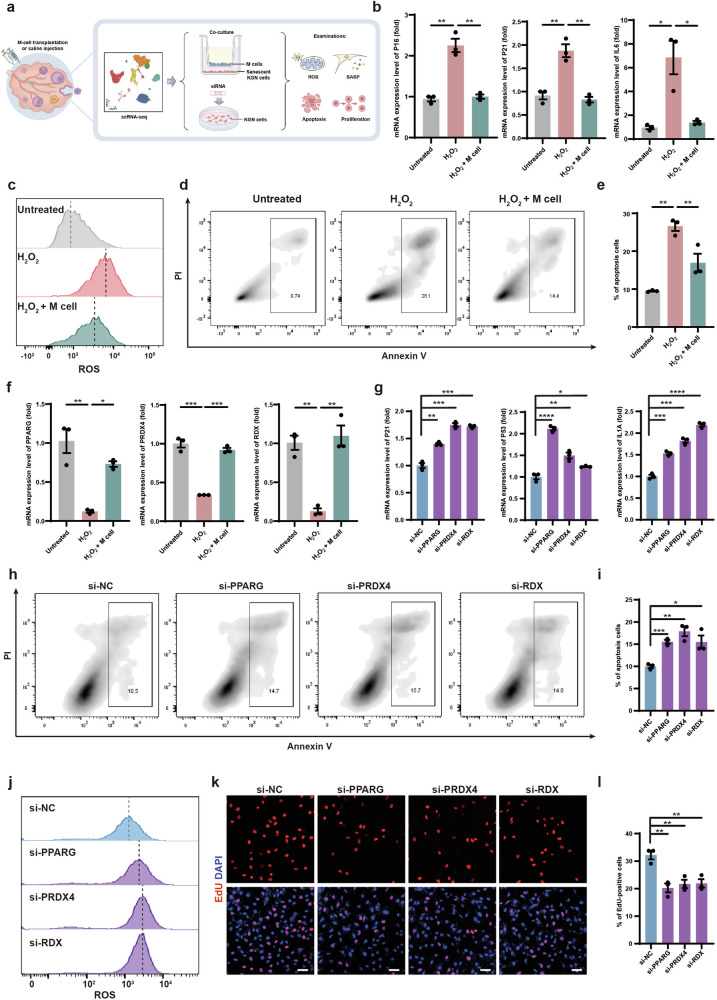
Effects of M cells on senescent GCs. **a** Schematic graphic showed in vitro validation of molecular mechanisms of M-cell transplantation for alleviating ovarian aging. **b** RT-qPCR analyses of *P16* (left panel), *P21* (middle panel), and *IL6* (right panel) expression levels in KGN cells of the untreated group, H_2_O_2_ group, and H_2_O_2_ + M cells group. *n* = 3, *P < 0.05, **P < 0.01 (two-tailed *t*-test). **c** Flow cytometry analysis showed cellular ROS levels in KGN cells of the untreated group, H_2_O_2_ group, and H_2_O_2_ + M cells group. **d** Flow cytometry analysis showed the cell apoptosis of KGN cells in the untreated group, H_2_O_2_ group, and H_2_O_2_ + M cells group. The frames denoted the proportions of apoptosis. **e** Bar graph illustrat**e**d the apoptotic rates of KGN cells in the untreated group, H_2_O_2_ group, and H_2_O_2_ + M cells group. *n* = 3. **P < 0.01 (two-way ANOVA). **f** RT-qPCR analyses of *PPARG* (left panel), *PRDX4* (middle panel), and *RDX* (right panel) expression level in KGN cells of the untreated group, H_2_O_2_ group, and H_2_O_2_ + M cells group. *n* = 3, *P < 0.05, **P < 0.01, ***P < 0.001 (two-way ANOVA). **g** RT-qPCR analyses of *P21* (left panel), *P53* (middle panel), and *IL1A* (right panel) in KGN cells after knockdown of *PPARG*, *PRDX4*, and *RDX*. *n* = 3, *P < 0.05, **P < 0.01, ***P < 0.001, ****P < 0.0001 (two-tailed *t*-test). **h** Flow cytometry analysis showed apoptosis of *PPARG*-, *PRDX4*-, and *RDX*-knockdown KGN cells. The frames denoted the proportions of apoptotic cells. **i** Bar graph illustrated the apoptotic rates of *PPARG*-, *PRDX4*-, and *RDX*-knockdown KGN cells. *n* = 3. *P < 0.05, **P < 0.01, ***P < 0.001 (two-tailed *t*-test). **j** Flow cytometry analysis showed cellular ROS levels in *PPARG*-, *PRDX4*-, and *RDX*-knockdown KGN cells. **k** Immunofluorescence analysis showed the EdU-positive KGN cells after *PPARG*, *PRDX4*, and *RDX* knockdown. Scale bars, 50 μm. **l** Bar graph showed the proportion of EdU-positive KGN cells after *PPARG*, *PRDX*4, and *RDX* knockdown. *n* = 3, **P < 0.01 (two-tailed *t*-test).

To investigate whether M cells could alleviate GC senescence in vitro, we co-cultured senescent KGN cells (a GC cell line) with M cells using transwell culture systems (Fig. 7a; Supplementary Fig. S10a). Firstly, to recapitulate the GC senescence features observed in naturally aging monkeys, we used hydrogen peroxide (H_2_O_2_)-treated KGN cells as an aging model^49,50^. After H_2_O_2_ treatment, KGN cells showed a significant increase in senescence-associated beta-galactosidase (SA-β-gal) activity (Supplementary Fig. S10b). Subsequently, M cells were seeded onto the top of the transwell membrane and co-cultured with senescent KGN cells (on the bottom layer) for 5 days (Supplementary Fig. S10a). We then examined the senescent indicators of KGN cells (untreated group, H_2_O_2_ group, H_2_O_2_ + M cells group). Senescent KGN cells exhibit considerably higher expression levels of the cell senescence markers *P16* and *P21*^51^, as well as the pro-inflammatory cytokine *IL6*, which is also a component of the senescence-associated secretory phenotype (SASP)^52^. However, these expression levels decreased upon co-culturing with M cells (Fig. 7b). To investigate whether M cells reduce oxidative damage and apoptosis in granulosa cells, we examined the ROS levels by the DCFH-DA assay and assessed apoptosis by Annexin V-PI apoptosis assay in KGN cells (Fig. 7c–e). We found that ROS levels and the proportion of apoptotic cells were significantly decreased in senescent KGN cells after co-culturing with M cells (Fig. 7c–e). These results suggested that M cells can inhibit oxidative damage and apoptosis in senescent granulosa cells.

Highly expressed genes of GCs in the treated group related to anti-inflammation, anti-oxidative damage, and proliferation, which were identified in the above scRNA-seq analysis, were *PPARG*, *PRDX4*, and *RDX*, respectively (Fig. 6f, g; Supplementary Fig. S10c–e). *PPARG* is a nuclear receptor that regulates metabolic processes, such as lipid uptake and oxidation and inhibits inflammation^53,54^. *PRDX4* is a vital endoplasmic reticulum-resident anti-oxidant in cells^55^. *RDX* is a membrane–cytoskeletal crosslinker in actin-rich cell surface structures and is essential for cell motility, adhesion, and proliferation^56,57^. Consistent with the scRNA-seq data, these protein levels were increased in the GCs of the treated group (Supplementary Fig. S10f–h). To investigate whether M cells could promote the expression of these genes in senescent granulosa cells in vitro, we analyzed the expression levels of *PPARG*, *PRDX4*, and *RDX* and found that their expression levels were significantly decreased in senescent KGN cells and could be rescued by co-culturing with M cells (Fig. 7f). To further explore the roles of these genes in granulosa cell senescence, we individually knocked down *PPARG*, *PRDX4*, and *RDX* in KGN cells by small interfering RNAs (siRNAs). Knockdown efficiencies were examined by quantitative reverse transcription PCR (RT-qPCR) (Supplementary Fig. S11a). After the knockdown of these genes, the expression levels of the *P21*, *P53*, and *IL1A* were significantly increased in KGN cells (Fig. 7g; Supplementary Fig. S11b, c). We also examined apoptosis and ROS levels in *PPARG-*, *PRDX4-*, and *RDX-*knockdown KGN cells and found significant increases in apoptosis rates and ROS levels (Fig. 7h–j). These results indicated that these genes may execute anti-inflammation, anti-oxidation, and anti-apoptosis effects in KGN cells. To evaluate the potential functions of these genes in regulating cell proliferation, we performed EdU staining in *PPARG*-, *PRDX4*-, and *RDX*-knockdown KGN cells and found significant decreases in the proportion of EdU-positive KGN cells (Fig. 7k, l). The Cell Counting Kit-8 (CCK8) assay also supported these findings (Supplementary Fig. S11d). These results indicated that *PPARG*, *PRDX4*, and *RDX* promoted the proliferation of senescent KGN cells.

**Fig. 8:**
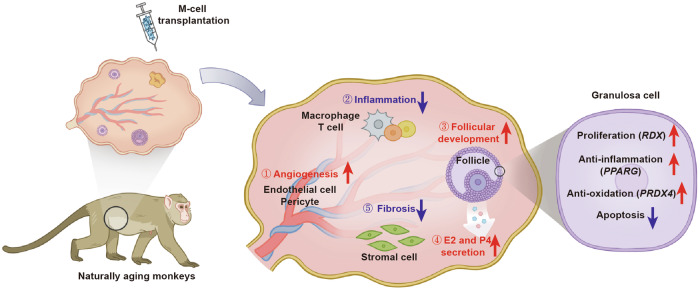
The possible mechanisms underlying the beneficial effects of M-cell therapy. Schematic diagram showed that M cells may ameliorate ovarian aging partially by decreasing the inflammation and fibrosis, as well as promoting the angiogenesis, follicular development, and sex hormone secretion.

Taken together, M cells may ameliorate ovarian aging partially by promoting the expression of *PPARG*, *PRDX4*, and *RDX* to decrease oxidative damage, apoptosis, and inflammation, and increase proliferation in senescent GCs (Fig. 8).

## Discussion

Physiological ovarian aging is an inevitable process that affects female reproduction and can reduce life quality. Unfortunately, there is currently no effective treatment available to fundamentally restore ovarian function and improve fertility. In this study, we first counted the number of primordial follicles in perimenopausal Chinese women’s ovaries and validated that there is a reserve of primordial follicles that possibly could be activated by stem-cell therapy. We then utilized perimenopausal cynomolgus monkeys as a non-human primate model to evaluate the safety and efficacy of M-cell transplantation in alleviating natural ovarian aging. We conducted in situ M-cell transplantation (treated group) or saline injection (control group) into the ovaries laparoscopically, followed by an 8-month follow-up. We found that M-cell therapy decreased inflammation, fibrosis, oxidative damage, and apoptosis, and increased the secretion of sex hormones in perimenopausal ovaries. Moreover, one of the aging cynomolgus monkeys with M-cell transplantation was conceived and gave birth to a healthy baby monkey. Together with the results from the scRNA-seq analysis, which depicted molecular mechanisms toward improving ovarian function, our findings provided evidence that M-cell administration enhanced the recovery of ovarian function.

Women are born with a fixed stock of ovarian follicles that serve their reproductive needs throughout their lifetime^3^. The ovarian reserve is steadily depleted with aging until the complete attrition which leads to menopause. Age at menopause shows variation from 44.6 to 52 y across different geographic regions^8^. Among these, Chinese women typically reach menopause at ~48.6 y^11^, while later menopause is shown in females from Europe, Australia, Canada, and the USA (48.4–52 y, 51.2 y, 49.1 y, and 51 y respectively)^8^. Women begin to experience perimenopausal vasomotor-related symptoms due to a sharp decline in hormone levels 1–3 years before their last menstruation^58^, and the average age for Chinese women entering this period is 46 y^10^. The age-related decrease of follicles dictates the onset of cycle irregularity and the cessation of menses^5^. It is a common concern whether any follicles remain in perimenopausal ovaries, and if so, how many and whether these follicles could be reactivated and developed to restore ovarian function? However, there has not yet been a direct measurement of the follicles representing the Chinese women’s ovarian reserve at perimenopause. The exploration of the dynamic ovarian reserve of Chinese women during the perimenopausal period will enhance our understanding of the ovarian reserve in this period, and further demonstrate the feasibility of stem-cell therapy against ovarian aging. We collected 28 ovaries and determined the numbers of primordial and primary follicles using the method we previously developed^1^. We found that Chinese women have an average of 11,098 primordial follicles per ovary at 35–39 y, which declined to 6728 at 40–44 y. The number was further reduced to 1019 at 45–49 y and 151 at 50–52 y, indicating the sharp decrease happened when women entered 45–49 y. Furthermore, we have also found that the ovaries of perimenopausal women still contained growing follicles even at 49 y, suggesting that primordial follicles could potentially be activated and developed into growing follicles at this stage. As women approach menopause at ~45–47 y, the ovaries still contain more than one thousand primordial follicles, highlighting the feasibility of restoring ovarian function and improving the life quality of perimenopausal women by facilitating the development of the remaining primordial follicles.

The number of MSCs isolated from adult or perinatal tissues is limited and the cells are heterogeneous. Besides, these MSCs are prone to senescence during in vitro passages, which further limits their potential in stem-cell-based therapy. M cells used in this study are derived from hESCs and resemble MSCs as they fulfill all of the criteria of MSCs^39^. Furthermore, M cells could be sufficiently expanded in vitro without limitation or undergoing senescence. After M-cell transplantation, we found the amelioration of ovarian functions in aging monkeys. We supposed that the therapeutic potential of M cells is strongly influenced by microenvironmental stimuli, where M cells secrete growth factors, cytokines, and chemokines (including HGF, vEGF, bFGF, EGF, IGF-1, etc.) with local paracrine effects^59,60^. The secretome is tightly associated with proliferation, angiogenesis, immunoregulation, and anti-fibrosis effects^59–62^. Similarly, through scRNA-seq in this study, we observed an increase in genes associated with cell proliferation and angiogenesis in the ovaries after M-cell injection, along with a reduction in genes related to immune response and fibrosis (Fig. 6g, h; Supplementary Fig. S9c–e). Follicular development is accompanied by angiogenesis and proliferation of granulosa cells and theca cells^63^. After M-cell transplantation, the proportions of GCs, TCs, endothelial cells, and pericytes in ovaries were increased, suggesting that M cells promoted follicle development, sex hormone secretion, and angiogenesis (Fig. 8). Accordantly, we found the M cell-treated monkeys presented ameliorated hormone levels, their ovaries showed more growing follicles, as well as higher levels of proliferation and lower levels of fibrosis, correlated well with the scRNA-seq analysis. Moreover, we co-cultured the senescent KGN cells with M cells by transwell culture systems and found that M cells promoted the expression of *PPARG*, *PRDX4*, and *RDX* and the senescent KGN cells showed a reduction in apoptosis and oxidative damage, along with a decreased expression of aging and inflammation-related factors (Fig. 7b). The results further indicated that M cells ameliorated the microenvironment and improved ovarian function via anti-oxidative, anti-apoptotic, and anti-inflammatory effects.

In conclusion, we deciphered the ovarian reserve of Chinese women at perimenopause and restored the ovarian function of naturally aging primates by M-cell transplantation. Furthermore, our results elucidated that M cells reduced inflammation, fibrosis, oxidative damage, and apoptosis and promoted angiogenesis and follicle development in the naturally aging ovaries. Our findings provided promise for the clinical application of M cells in ameliorating female reproductive aging and improving fecundity (Fig. 8).

## Materials and methods

### Ethical statement

The collection of human ovaries was approved by the Human Ethics Committees of the First Affiliated Hospital of Zhengzhou University (2020-KY-221), the Peking Union Medical College Hospital (JS-2815), and the Hospital for Reproductive Medicine Affiliated to Shandong University ([2022] LSZ 01) and was conducted in compliance with approved institutional guidelines. The study has been performed following the ethical standards laid down in the 1964 Declaration of Helsinki and its later amendments or comparable ethical standards.

The experiments involving cynomolgus monkeys were conducted according to the Principles for the Ethical Treatment of Non-Human Primates and were approved in advance by the Institutional Animal Care and Use Committee of the Institute of Zoology (IOZ-IACUC-2021-178). All applicable institutional and national guidelines for the care and use of animals were followed in this study.

All experiments were conducted according to the standard operating procedure of the Institute of Zoology.

### Source of human ovaries

Physiologically normal human ovaries were obtained from 26 women. These women suffered from uterine diseases and underwent hysterectomy and bilateral salpingo-oophorectomy to prevent poor prognosis caused by estradiol secretion and the risk of metastatic lesions spreading to the ovaries after surgery. The exclusion criteria for donors referred to a previous study^40^. Briefly, donors previously exposed to chemotherapy, or with previous ovarian surgery were excluded from the study. Additionally, ovarian pathology such as endometriomas and cystic masses of the ovary >2 cm was excluded from the study. Specimens with gross or microscopic evidence of ovarian pathology were also excluded from the study. Medical records, surgical pathology reports, and operative reports were reviewed. The ovary of a 23-year-old woman, who died of brain herniation, was donated from Peking Union Medical College Hospital.

### Source of cynomolgus monkeys

All cynomolgus monkeys (*Macaca fascicularis*) were of Southeast Asian origin. The animals were maintained at ~25 °C on 12 h light–12 h dark cycle and raised at the Xieerxin Biology Resource with the accreditation of the laboratory animal care facility of the Institute of Zoology. All animals were fed a commercial diet twice daily while vegetables and fruits once daily, with tap water ad libitum, under careful veterinary oversight. Before the experiment, none of the animals had a clinical or experimental history that would affect physiological aging or increase their susceptibility to diseases. Cynomolgus monkeys were anesthetized with ketamine (10–12 mg/kg) before surgery. A total of 10 cynomolgus monkeys aged 18–23 y were selected. The ovarian sections of young monkeys (3 y) were from the remaining samples of our previous study^1^.

### Cell lines

M cells were generated by the National Stem Cell Resource Center, Institute of Zoology, Chinese Academy of Sciences as described previously^39^. Firstly, clinical human embryonic stem cells (hESCs, Q-CTS-hESC-2)^64^ were dissociated into small clumps to form human embryoid bodies (hEBs) for 5 days. Subsequently, hEBs were transferred onto plates and cultured for additional 14 days. The hEBs outgrowth cells were dissociated and passaged continuously in M-cell Medium consisting of α-MEM (Gibco, 12561-049) supplemented with 5% KOSR (Gibco, A3020902), 1% Ultroser G (Pall corporation, 15950-017), 1× L-glutamine (Gibco, A12860-01), 1× NEAA (Gibco, 11140050), 5 ng/mL bFGF (R&D systems, 233-FB) and 5 ng/mL TGF-β (Peprotech, 96-100-21-10). After 5 passages, ~4 weeks, M cells were harvested. They displayed a fibroblastic morphology and expressed canonical MSC-specific surface markers.

KGN cells, a human granulosa tumor cell line, were cultured as previously described in DMEM/F12 medium (GIBCO, 11320033) supplemented with 10% fetal bovine serum (FBS) (GIBCO, 10091-148), 100 U/mL penicillin, and 100 μg/mL streptomycin (GIBCO, 15070063) at 37 °C in 5% CO_2_. All the cell cultures tested negative for mycoplasma contamination.

### Transwell culture system

KGN cells were plated in the lower chambers of transwell plates (Corning, 3450), and then were treated with 100 µM H_2_O_2_ for 24 h, followed by 45 µM H_2_O_2_ for 24 h to induce cellular senescence. Subsequently, M cells were seeded into the upper chambers of the transwell plates. After 5 days, the KGN cells in the lower chamber were harvested for apoptosis, ROS, and RT-qPCR analyses.

### Follicle identification and counting

Ovaries were obtained right after surgical removal. In the case of elective surgical removal, a small part of the ovary was removed and prepared separately for review by a surgical pathologist. The remainder of the ovary was then re-weighed. The difference in weights recorded was used to determine the fraction of the whole ovary that was available for use in this study (the first fraction, f1). Each ovary was first cut into 1-mm slabs perpendicular to the long axis using a slicer designed by our lab^1^. Approximately one-third of the slabs were selected out of the total generated slabs (the second fraction, f2) using systematic random sampling rules. Systematic random sampling rules require that an interval for sampling be set, a random position within that interval be determined for the initial sample, and then sequential samples be selected at that fixed interval. The selected slabs were embedded in paraffin wax (Leica, 39601006) following immersion through a graded series of alcohol solutions (70%, 80%, 90%, 100%, and 100%). The slabs were then cut into 5-μm-thick sections using a rotary microtome (Leica, Leica 2135). Subsequently, five serial sections from every 50 sections (the third fraction, f3) were collected from each slab and stained with H&E. The five H&E-stained serial sections were scanned using a PerkinElmer Vectra Polaris (Leica, Leica Aperio VESA8).

Follicles were classified according to the morphological criteria described by Charleston^40^. Primordial follicles were defined as those containing a single layer of flattened granulosa cells while primary follicles were defined as those containing a single layer of cuboidal granulosa cells without any flattened granulosa cells. Primordial and primary follicles were counted when a clearly defined oocyte nucleolus was observed. Follicles that had already been counted were excluded from the subsequent adjacent section.

Raw counts (Q) for five serial sections of follicles were then converted to an estimate of the total number (N) in the entire ovary by the following equation (where Q is the raw count for each serial set of sections):

$$\text{N}=\sum \text{Q}\times 1/\left(\text{f}1\times \text{f}2\times \text{f}3\right)$$

Secondary follicles were defined as having an enlarged oocyte surrounded by at least a partial or complete second layer of cuboidal GCs but no more than four complete layers of cuboidal GCs. Antral follicles were characterized by the presence of areas of follicular fluid (antrum) or a single large antral space. As the volume of secondary follicles is significantly larger than that of primordial and primary follicles, the secondary follicles appear in more tissue sections and tend to be overcounted when using the same follicle counting method for primordial and primary follicles. Therefore, we compared the relative number of secondary follicles in different female ovaries by selecting 1/3 slabs and counting follicles in one out of every 50 serial sections.

For growing follicle counting of monkeys, one ovary per group was analyzed, and 3 sections were taken from each ovary, with a 50-section interval between each selected section. The entire section was used to count the number of growing follicles.

### Ultrasound evaluation of ovaries and uteruses

The ultrasonic examination was performed before and after treatment to evaluate the size of the ovary, and the endometrial thickness. Monkeys were examined in the supine position, using CX50 compact Xtreme ultrasound system (Philips Ultrasound, Inc.). The mean diameter of the ovaries was calculated as follows: D = [L (maximal length) + W (maximal width)] ∕ 2.

### M-cell transplantation and saline injection

Cynomolgus monkeys were anesthetized with ketamine (10–12 mg/kg) before surgery. M cells (100 μL per ovary, 5 × 10^6^ cells, treated group) or 0.9% saline (100 μL per ovary, control group) were injected into the ovary of monkeys by laparoscopy. The solution was injected into the ovary using 25-g needles (World Precision Instruments, Inc, 3030217) by two senior medical physicians.

### Superovulation, ICSI, and embryo culture

As described in a previous study^65^, female cynomolgus monkeys received twice daily intramuscular injections of rhFSH (GONAL-F, Merck Serono) for 8 days, beginning at days 1–3 of the menstrual cycle, then received injection of rhCG (OVIDREL, Merck Serono) on day 9. Oocytes were aspirated laparoscopically at 32–35 h after rhCG administration. MII oocytes (with the first polar body) were kept in hamster embryo culture medium-10 (HECM-10) until ICSI. The fertilized embryos were then cultured in 50-µL drops of HECM-10 containing 10% FBS at 37 °C in a humidified atmosphere of 5% CO_2_, with the medium changed every other day.

### H&E staining

The ovarian sections were deparaffinized and rehydrated using a graded alcohol series (100%, 95%, 85%, and 75%). After a brief wash in distilled water, the slides were incubated in a hematoxylin solution for 4 min. The sections were then washed with running tap water for 10 min to remove excess hematoxylin. Next, the sections were incubated in 1% acid alcohol for 5 s and washed with running tap water for 3 min. This step was followed by incubation in eosin counterstain for 8 min, dehydration in a graded alcohol series (95%, 100%, and 100%), and immersion in xylene. Finally, the slides were sealed with coverslips using Cytoseal-60 (Stephens Scientific, USA).

### Immunostaining of ovarian tissues and KGN cells

The ovarian sections were deparaffinized and rehydrated using 100%, 95%, 85%, and 75% alcohol. After washing in distilled water, antigen retrieval was conducted by boiling the slides for 15 min in Tris-EDTA buffer and cooling the slides for 2 h. After washing three times with phosphate-buffered saline (PBS), the sections were blocked with 3% bovine serum albumin (BSA, Sigma–Aldrich, A1933) for 1 h at 25 °C. Then, the sections were incubated with primary antibodies at 4 °C overnight. After washing thrice with PBS, the sections were incubated with the corresponding secondary antibodies and DAPI (Thermo Fisher Scientific, USA, D3571) for 1 h at 25 °C. The slides were sealed with coverslips using Cytoseal-60 (Stephens Scientific, USA). A laser scanning confocal microscope (Carl Zeiss, Germany, LSM 880) was used to capture images. Three sections were selected per ovary, with each section spaced 50 slices apart and 5 regions were randomly selected for each section. The assessors were blinded to the treatment group when performing the image capture.

The KGN cells were fixed in 4% PFA for 30 min and permeabilized in 0.5% Triton X-100 (Sigma) for 30 min. After being washed in 0.1% BSA three times, the KGN cells were blocked with 5% BSA in PBS solution for 1 h and incubated with primary antibodies overnight at 4 °C. The KGN were then incubated with corresponding secondary antibodies for 1 h and counterstained with DAPI for 15 min. The images were captured using Leica Stellaris.

The primary antibodies included: Rabbit anti-γ H2A.X (Abcam, ab81299, 1:200), Rabbit anti-Ki67 (Abcam, ab15580; 1:200), Rabbit anti-PPARG (Cell Signaling Technology, 2345t; 1:200), Rabbit anti-PRDX4 (Abcam, ab184167; 1:200), Rabbit anti-RDX (Abcam, ab52495; 1:200), Rabbit anti-P21 (Cell Signaling Technology, 2947 P, 1:200), Rabbit anti-P53 (Santa Cruz, sc-126, 1:200). The secondary antibodies included: Alexa Fluor 568 donkey anti-rabbit antibody (Thermo Fisher Scientific, A10042; 1:200), Alexa Fluor 568 donkey anti-mouse antibody (Thermo Fisher Scientific, A10037; 1:200).

### Masson’s trichrome staining of ovarian tissues

Fibrosis was detected by Masson’s trichrome staining kit (Solarbio, G1340). The ovarian sections were deparaffinized and rehydrated using 100%, 95%, 85%, and 75% alcohol. After washing in distilled water, the sections were stained with an iron hematoxylin working solution for 5 min. Next, the sections were washed with distilled water and incubated in an acid-alcohol solution for 5 s. The sections were then washed with tap water for 5 min. Next, the sections were stained in a ponceau-acid fuchsin solution for 8 min, rinsed in distilled water, and incubated in phosphotungstic acid for 1 min. The sections were then directly transferred (without rinsing) to an aniline blue solution and stained for 20 s. This step was followed by a brief rinse in distilled water and fixation in 1% acetic acid solution for 1 min. After washing in distilled water, the sections were dehydrated quickly through 95% alcohol and 100% alcohol and cleared in xylene. Finally, the sections were mounted on a resinous mounting medium. Fibrotic areas are indicated in the blue-stained regions. Red areas denote muscle fibers, cytoplasm, cellulose, keratin, and red blood cells. Quantification of the fibrotic area was performed using ImageJ software (Softonic). Each picture was separated into different colors. Subsequently, an appropriate channel was chosen, and the appropriate threshold value was applied to all pictures. One ovary per group was analyzed, and 3 sections were taken from each ovary for Masson’s staining, with a 50-section interval between each selected section. The entire section was used to calculate the fibrosis area. Therefore, the fibrosis calculation formula is the fibrotic area /total area in the whole section × 100%.

### Sex hormone assays

Blood samples were collected from 10 cynomolgus monkeys aged 18–23 y. At the time of collection, serum specimens were aliquoted and stored at –80 °C until the sex hormone assays were performed. The serum levels of E2 (Roche, 06656021190) and P4 (Roche, 07092539190) were measured by Cobas6000 (Roche). Before and after 3-, 6-, and 8-month treatment, we collected blood samples every 4 days for 6 weeks. Then we analyzed the E2 and P4 levels at day 1 (corresponding to the early follicular phase) of the menstrual cycle because the sex hormone levels were relatively stable during this period^45^.

### CCK8 assay

The experiments were performed following the instructions of Cell Counting Kit-8 (LABLEAD, China). Briefly, a total of 2 × 10^3^ viable cells were plated in each well of the 96-well plates (Corning). After incubation for the first 24 h, the viable cell number was then calculated every 24 h for three consecutive days. To determine the number of viable cells, the optical density value at 450 nm was detected with BioTex Power Wave XS (BioTex, USA).

### EdU assay

According to the manufacturer’s instructions (Ribobio, C10310-1), KGN cells were incubated with 5-ethynyl-20-deoxyuridine (EdU) for 2 h. After being fixed with 4% PFA for 30 min and permeabilized with 0.5% Triton X-100 for 10 min, the cells were incubated with 1× Apollo®567 reaction cocktail for 30 min. The cell nuclei were finally stained with DAPI for 10 min. The images were captured using Zeiss LSM 880.

### Beta-galactosidase staining

SA-β-gal staining was done using the SA-β-gal staining kit (Beyotime, C0602) according to the manufacturer’s instructions. Briefly, 1. Remove growth media from the cells. 2. Rinse the plate one time with 1× PBS. 3. Add 1 ml of Fixative Solution. Allow cells to fix for 10–15 min at room temperature. 4. Rinse the plate two times with 1× PBS. 5. Add 1 ml of the β-Galactosidase Staining Solution to each well. 6. Incubate the plate at 37 °C at least overnight in a dry incubator (without CO_2_). 7. While the β-galactosidase is still on the plate, check the cells under a microscope (200× total magnification) for the development of blue color and take images. 8. For long-term storage of the plates, remove the β-Galactosidase staining solution and overlay the cells with 70% glycerol.

### siRNA-mediated knockdown of *PPARG*, *PRDX4*, and *RDX*

*si-PPARG*, *si-PRDX4*, *si-RDX*, and non-targeting si-NC were transfected into KGN cells using jetPRIME Transfection Reagent (Polyplus, 114-01). At 48–96 h after transfection, cells were collected for downstream analyses. All siRNAs were synthesized by TsingkeBiotechnology Co., Ltd.

### Flow cytometry analysis

For the measurement of cellular ROS, cells were collected and stained with 10 μM DCFH-DA (Solarbio, D6470) for 30 min at room temperature, and then the signals were quantified in a BD LSRFortesa flow cytometer. For analysis of apoptosis, cells were collected freshly and stained with Annexin V-EGFP and PI using an Annexin V-EGFP Apoptosis Detection Kit (Beyotime, C1062M). Then, the apoptotic cells were quantified in a BD LSRFortesa flow cytometer.

For the characterization of M cells, cells were harvested and blocked with 2% BSA (Sigma–Aldrich, B2064) for 20 min at room temperature. Then, the cells were stained with fluorescein-conjugated antibodies for 40 min at room temperature in 1% BSA. After incubation, cells were washed 3 times with PBS and analyzed with MoFlo (Beckman, Brea, CA, USA) and associated software. The antibodies used for flow cytometry were as follows: PE-conjugated mouse anti-human CD29 (Biolegend, San Diego, CA, USA; 303004), PE-conjugated mouse anti-human CD73 (BD Biosciences, San Jose, CA, USA; 550257), PE-conjugated mouse anti-human CD90 (eBioscience, San Diego, CA, USA; 12-0909-42), PE-conjugated mouse anti-human CD105 (Biolegend, 323206), PE-conjugated mouse anti-human CD34 (BD Biosciences, 555822), PE-conjugated mouse anti-human CD45 (BD Biosciences, 560957), PE-conjugated mouse anti-human HLA − DR (BD Biosciences, 555561), and the PE-conjugated mouse IgG1 (BD Biosciences, 551436) as an isotype control.

### RT-qPCR

Total RNA was extracted from cells and tissues using TRIzol reagent (Invitrogen, 15596018). The RNA was reverse-transcribed into cDNA by reverse transcriptase (Vazyme, R333-01) and then subjected to an RT-qPCR assay using TB Green® Premix Ex Taq™ II (Tli RNaseH Plus) (TAKARA, RR820A). All quantified values were normalized to the endogenous *GAPDH* expression levels. The sequences of the primers used for RT-qPCR are listed in Supplementary Table S6.

### Preparation of single-cell suspensions for scRNA-seq

Single-cell isolation was performed based on a protocol described previously with modification^66^. In brief, the ‘clean’ ovaries were cut first into 1-mm thick pieces and then minced using a tissue section (Mcllwain Tissue Chopper, The Mickle Laboratory, Guildford, UK). The minces were transferred to a 50-mL conical tube and digested enzymatically in a medium containing α-MEM (Gibco, 12561-056) + 0.04 mg/mL Liberase DH (Roche, 5401054001) + 0.4 mg/mL DNase I (Sigma, DN25) + 1% penicillin/streptomycin (Gibco, 15140-122) for 60 min in a shaker (160 rpm) at 37 °C interspersed with 20–30 pipetting every 15 min. Digestion was terminated by α-MEM media with 10% HSA (Merck Millipore, 823022). After digestion, the cell suspension was filtered by a 40-µm strainer and centrifuged at 300× *g* for 5 min, followed by gentle removal of the supernatant. For removal of red blood cells, the cell pellet was treated with 3 mL of ACK (ammonium chloride-potassium) lysis buffer for 10 min at room temperature, and then, the treatment was terminated with 27 mL of PBS. The remaining cells were washed twice with PBS and centrifuged at 300× *g* for 5 min. For the single-cell suspension, the cell pellet was resuspended in PBS and adjusted to a concentration of 700–1200 cells/μL to ensure subsequent cell capture efficiency. Cell concentration and viability were measured with a Countess II automated cell counter (Thermo Fisher, AMQAX1000). The cells were then used for single-cell sequencing.

### scRNA-seq library construction and sequencing

According to the manufacturer’s guidelines, cells were loaded onto a 10x Genomics Chromium chip targeting an 8000–10,000 cell ranger. Subsequently, reverse transcription and library preparation were carried out using the 10x Genomics Single Cell v2 kit, following the established 10x Genomics protocol. The constructed libraries were then subjected to sequencing on an Illumina NovaSeq 6000 platform, employing two lanes to generate 150 paired-end reads.

### Preprocessing scRNA-seq data

The initial step involved the processing of raw sequencing reads using Cell Ranger (v3.1, 10x Genomics) with default parameters. To facilitate read alignment and quantification, a reference genome was constructed, following the recommended guidelines from 10x Genomics, utilizing the Macaca fascicularis_5.0 genome and annotations obtained from Ensemble (version 102). In summary, the raw reads were aligned against the reference genome and subjected to quality checks. Only uniquely mapped reads were considered for the unique molecular identifier (UMI) counting. Subsequently, gene expression levels were quantified for each observed barcode. Cells exhibiting a significantly lower RNA content were excluded from the subsequent analysis. The filtered feature barcode matrices, generated through Cell Ranger (v3.1, 10x Genomics), were employed for downstream analyses.

### Identification of cell populations in scRNA-seq data

In this study, gene expression data were processed using the R package Seurat (v4.0.4)^66^. The raw counts were normalized by scaling to 10,000, and expression levels were defined as log_2_ (normalized counts + 1). As part of quality control, cells failing to meet the following criteria were filtered out: (1) the number of detected UMIs was at least 1000; (2) the number of detected genes was at least 500; and (3) the log-transformed ratio of detected genes to detected UMIs was higher than 0.8. Consensus variable features among samples were first identified as previously reported. For each sample, 3000 features were selected, and a consensus list of 3000 features was generated based on overlaps across samples. To remove potential sample/batch effects, Harmony (v0.1.1) was employed for dimension reduction with these consensus features. Subsequently, potential doublets in individual samples were identified using the Python package Scrublet (v0.2.3). Doublets along with cells in clusters exhibiting a high fraction of doublets were filtered out. After stringent quality control, a total of 26,862 cells remained, and the second round of clustering was performed using the new consensus features generated by Seurat.

### Identification of DEGs and GO enrichment analysis

The DEGs were identified using the FindAllMarkers or FindMarkers function in Seurat. Genes with an adjusted *P*-value less than 0.05 were considered DEGs. GO analysis was performed by using the R package clusterProfiler (v4.0.5) with the annotation R package org.Hs.eg.db (v3.13.0).

### Transcriptional regulatory network analysis

The pySCENIC Python package (v0.11.2) was adopted to infer the transcriptional regulatory network for GCs and SCs. Briefly, raw counts and a list of known TFs for humans were used as input for network inference (GRNBoost2), followed by the generation of candidate regulons (cisTarget). The activity score of candidate regulons in the individual cell was quantified using AUCell. Activity scores were averaged, scaled, and visualized using a heatmap. The network of the regulon and its targets was visualized using Cytoscape (v3.8.0).

### Inference of cell–cell interactions based on scRNA-seq data

For inference of cell–cell interactions between GCs and SCs, the CellPhoneDB Python package (v2.14.0) was adopted.

### Statistical analyses

The experimental data were statistically analyzed using one-way ANOVA, two-way ANOVA, or *t*-test to compare differences between different groups using PRISM software (GraphPad 9 Software). A *P*-value < 0.05 was considered statistically significant. In all figures, one, two, three, and four asterisks indicate *P* < 0.05, *P* < 0.01, *P* < 0.001, and *P* < 0.0001, respectively.

## Supplementary information

Supplementary information

Supplementary Table S7

## Data Availability

The sequencing data generated in this study have been deposited in Gene Expression Omnibus (GEO) under accession GSE242950.
