# Supplementary material for: Stem cell transplantation extends the reproductive life span of naturally aging cynomolgus monkeys
Source: Cell Discov. 2024 Nov 5;10:111. doi: 10.1038/s41421-024-00726-4 (PMC11535534; doi:10.1038/s41421-024-00726-4)
Supplement: Supplementary file 1 — Supplementary information [file 41421_2024_726_MOESM1_ESM.pdf]

# Stem cell transplantation extends the reproductive life span of naturally aging cynomolgus monkeys

Long Yan[1,9,10,11,†], Wan Tu[1,9,10,11,†], Xuehan Zhao[1,2,13,†], Haifeng Wan[1,9,10,11,†], Jiaqi Wu[1,2,13,†], Yan Zhao[1,9,10,11,†], Jun Wu[1,10,11,12,†], Yingpu Sun[3,†], Lan Zhu[4,†], Yingying Qin[5,8,†], Linli Hu[3,†], Hua Yang[4,†], Qiong Ke[6,†], Wenzhe Zhang[5,8,14,†], Wei Luo[5,8,14,†], Zhenyu Xiao[11,15,†], Xueyu Chen[7,†], Qiqian Wu[1,9,10,11], Beijia He[1,9,10,11], Man Teng[1,9,10,11], Shanjun Dai[3], Jinglei Zhai[1,9,10,11], Hao Wu[1,10,11], Xiaokui Yang[2,13,16], Fan Guo[1,9,10,11,16], and Hongmei Wang[1,9,10,11,*]

1. Key Laboratory of Organ Regeneration and Reconstruction, State Key Laboratory of Stem Cell and Reproductive Biology, Institute of Zoology, Chinese Academy of Sciences, 100101 Beijing, China.
2. Department of Human Reproductive Medicine, Beijing Obstetrics and Gynecology Hospital, Capital Medical University, 100026 Beijing, China.
3. Center for Reproductive Medicine, Henan Key Laboratory of Reproduction and Genetics, The First Affiliated Hospital of Zhengzhou University, 450001 Zhengzhou, China.
4. Department of Obstetrics and Gynecology, National Clinical Research Center for Obstetric & Gynecologic Diseases, Peking Union Medical College Hospital, Chinese Academy of Medical Sciences & Peking Union Medical College, 100730 Beijing, China.
5. Reproductive Hospital Affiliated to Shandong University, 250001 Jinan, China.
6. The First Affiliated Hospital, Sun Yat-sen University, 510080 Guangzhou, China.
7. Laboratory of Neonatology, Department of Neonatology, Affiliated Shenzhen Maternity & Child Healthcare Hospital, Southern Medical University, 518028 Shenzhen, China.
8. State Key Laboratory of Reproductive Medicine and Offspring Health, 250012 Jinan, China.
9. University of Chinese Academy of Sciences, 100049 Beijing, China.
10. Institute for Stem Cell and Regeneration, Chinese Academy of Sciences, 100101 Beijing, China.
11. Beijing Institute for Stem Cell and Regenerative Medicine, 100101 Beijing, China.
12. National Stem Cell Resource Center, Chinese Academy of Sciences, 100101 Beijing, China.
13. Beijing Maternal and Child Health Care Hospital, 100026 Beijing, China.
14. Department of Obstetrics and Gynecology, Shandong Provincial Hospital, Affiliated to Shandong First Medical University, 250021 Jinan, China.

15. School of Life Science, Beijing Institute of Technology, 100081 Beijing, China.
16. Senior author
† These authors contributed equally to this article.
* Corresponding authors.
**Correspondence:**
Hongmei Wang, State Key Laboratory of Stem Cell and Reproductive Biology, Institute of Zoology, Chinese Academy of Sciences 100101 Beijing, China. Emails: wanghm@ioz.ac.cn

**Running title:**

Stem cell therapy ameliorates naturally ovarian aging

# Supplementary Figures

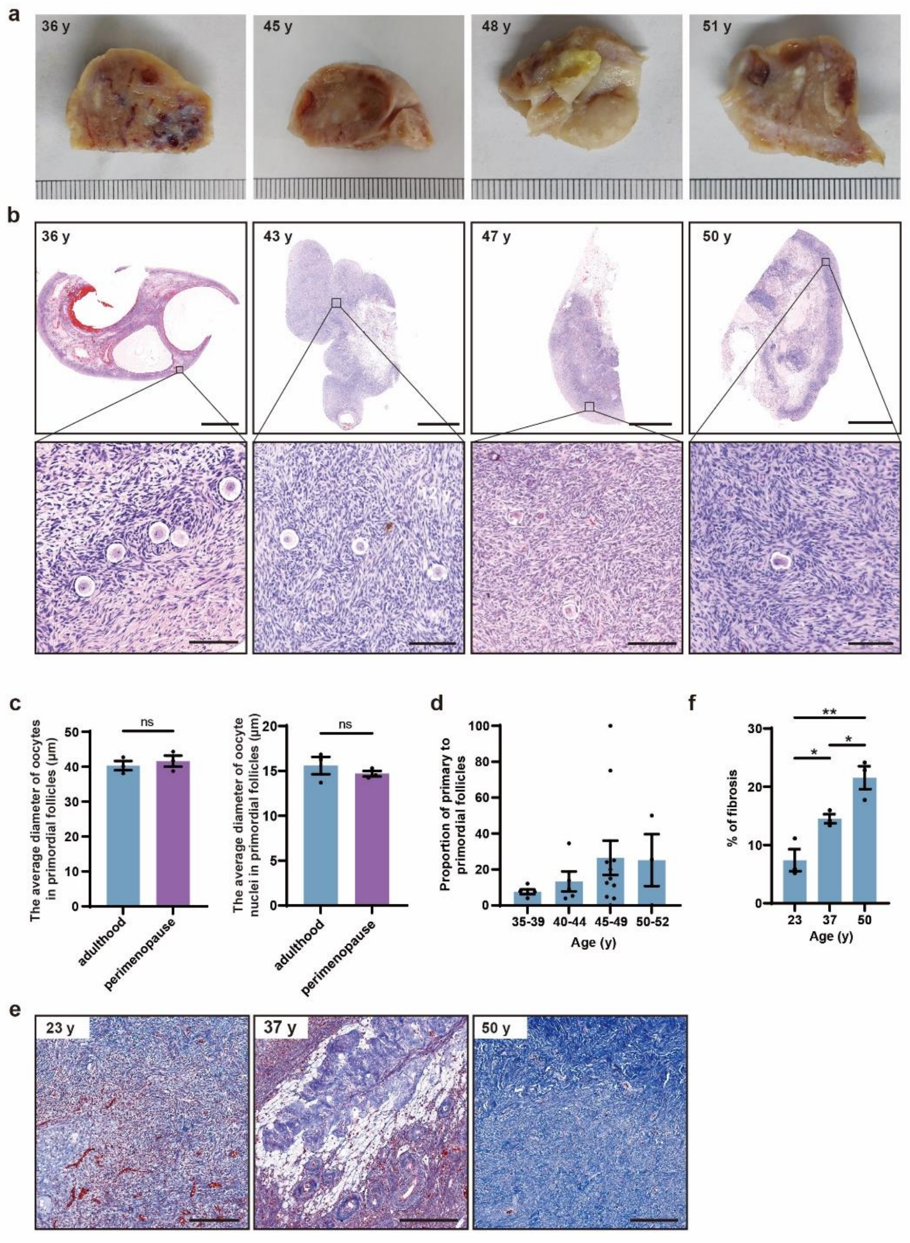


**Supplementary Fig. S1 Collection of ovaries from perimenopausal Chinese women and their morphological characteristics.**

a. Photographs of ovaries obtained from 36 years old (y), 45 y, 48 y, and 51 y women.

b. H&E-stained images showed the morphology of 36 y, 43 y, 47 y, and 50 y women's ovaries. Scale bars, 3 mm and 100 μm (magnified regions).

c. Bar graphs illustrated the average diameters of oocytes and oocyte nuclei in primordial follicles of women in adulthood (n = 3) and perimenopausal (n = 3) stages. ns, no significance, two-tailed t-test.

d. Bar graph illustrated the proportion of primary to primordial follicles in the 35-52 y women's ovaries. n (35-39 y) = 5, n (40-44 y) = 5, n (45-49 y) = 11, n (50-52 y) = 3. Cases 23 (50 y) and 26 (52 y) were not included in the data because no follicles were found in their ovaries.

e. Masson's trichrome staining showed fibrosis in ovarian sections of 23 y, 37 y, and 50 y women's ovaries. Blue areas denoted collagen fibers (fibrosis). Red areas denoted muscle fibers and cytoplasm. Scale bars: 200 μm.

f. Bar graph illustrated fibrosis areas in the ovaries of 23 y, 37 y, and 50 y women. n = 3 sections, $*P < 0.05$ and $**P < 0.01$ (one-way ANOVA).

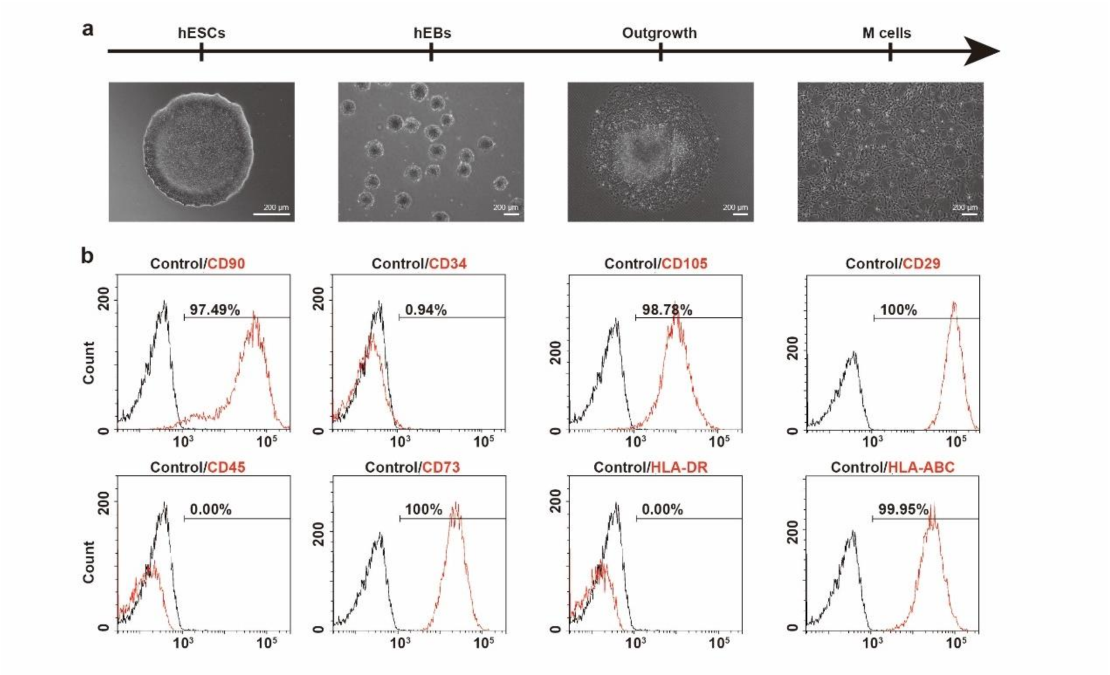


**Supplementary Fig. S2 Characteristics of M-cells derived from hESCs.**

a. Typical cell morphology during hESCs differentiation of M cells. Scale bars, 200 µm.

b. M cells' expression of MSC-specific surface markers was determined by flow cytometry.

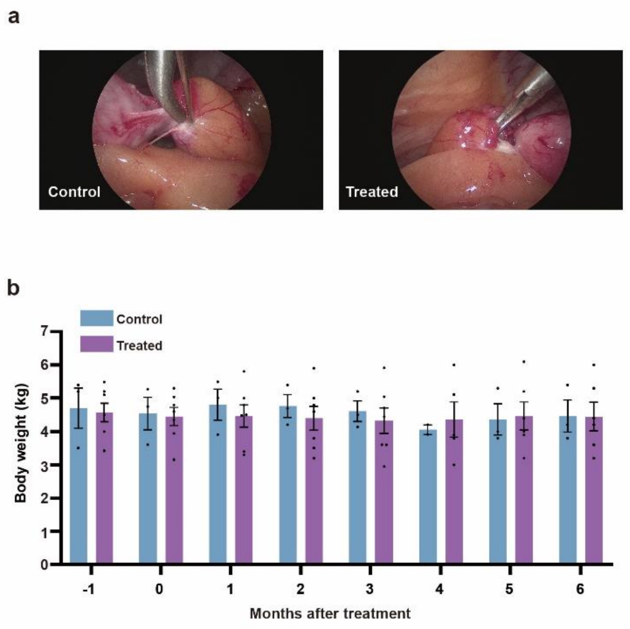


**Supplementary Fig. S3**

a. Laparoscopic images showed saline injection (control, left panel) and M-cell transplantation (treated, right panel) in monkey ovaries.

b. Bar graph illustrated the body weights of control and treated monkeys before treatment and after 0-, 1-, 2-, 3-, 4-, 5-, and 6-month treatment. n (control) = 3, n (treated) = 7 monkeys.

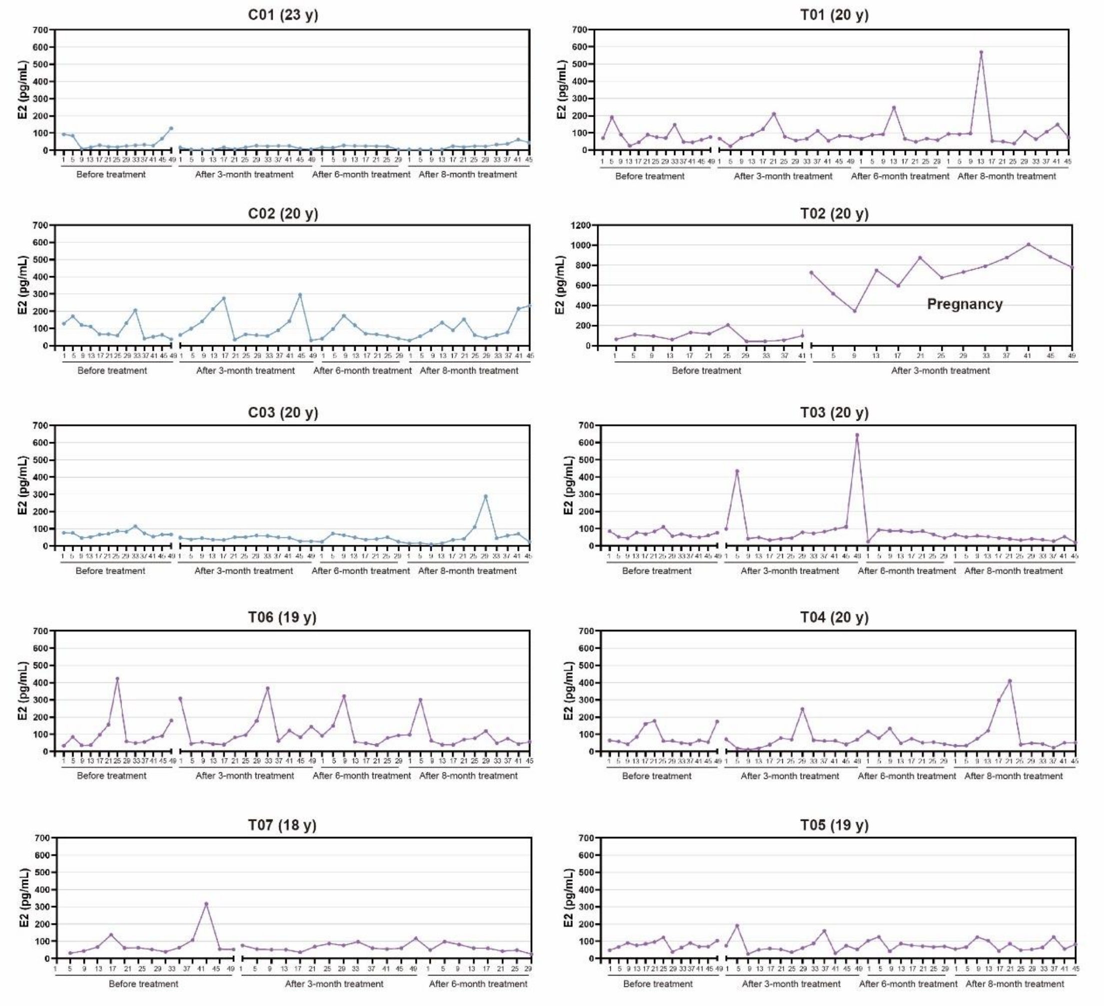


**Supplementary Fig. S4 M-cell transplantation recovered the E2 levels.**

Line plots illustrated the E2 levels of control (C01-C03) and treated (T01-T07) monkeys before treatment and after 3-, 6- and 8-month treatment.

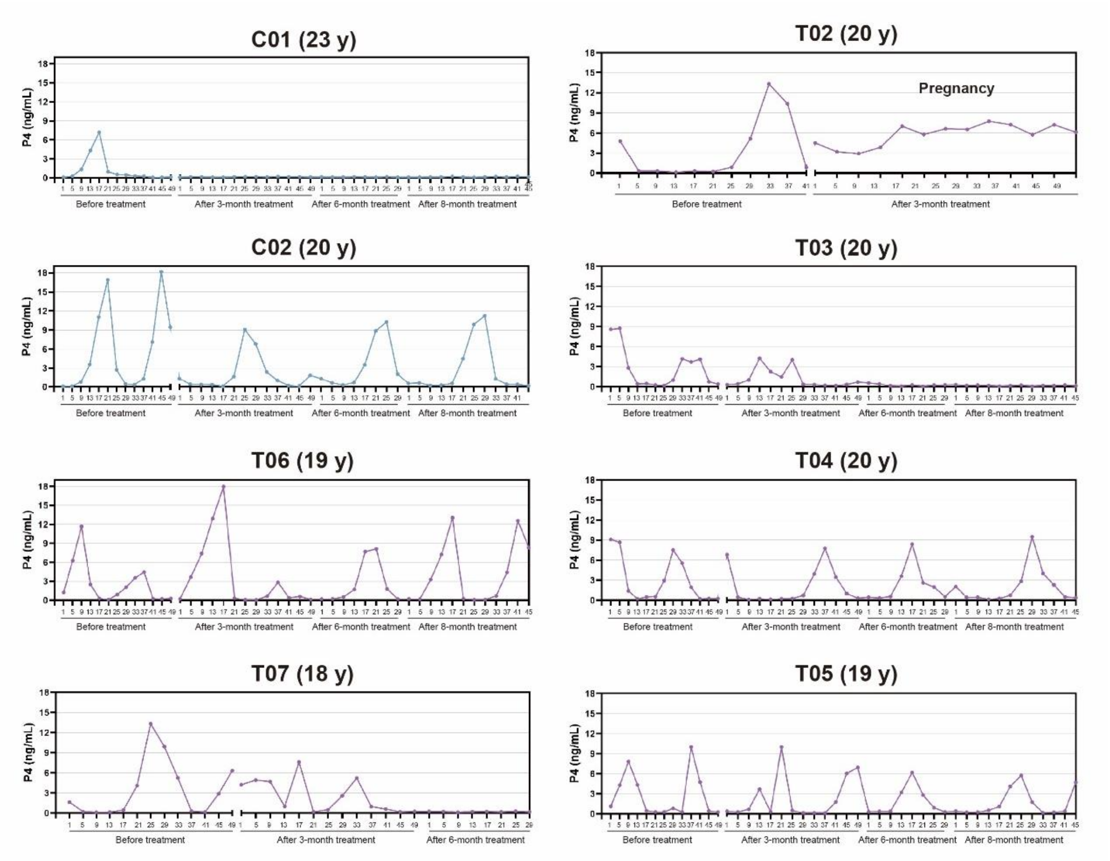


**Supplementary Fig. S5 M-cell transplantation improved the P4 levels.**
Line plots illustrated the P4 levels of the control (C01 and C02) and treated (T02, T03, T04, T05, T06, and T07) monkeys (except the pregnant monkey T02) before treatment and after 3-, 6- and 8-month treatment.

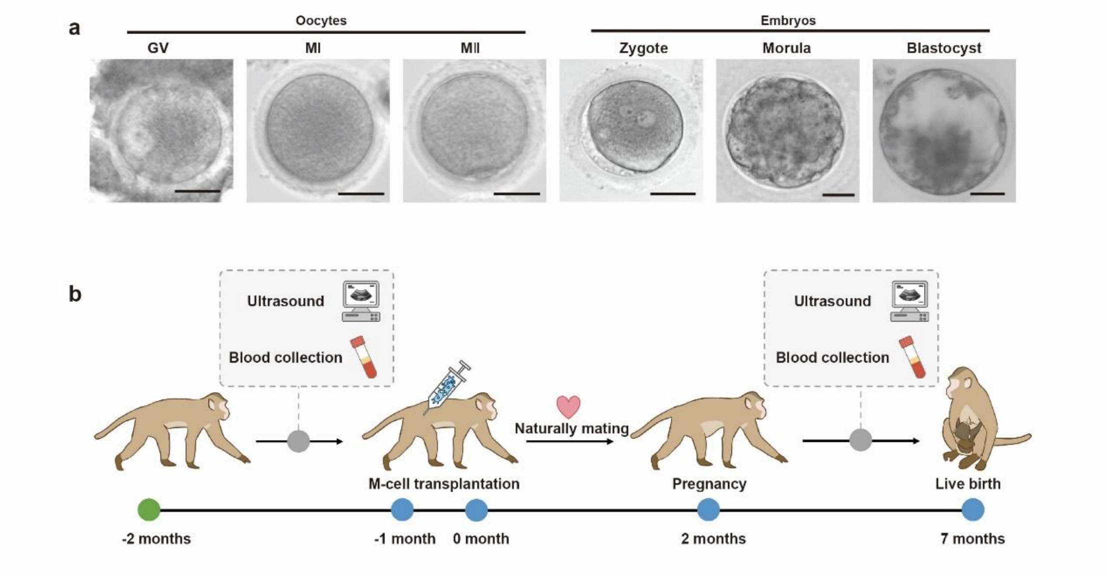


**Supplementary Fig. S6 M-cell transplantation improved the fertility potential of naturally aging monkeys.**

a. Phase-contrast micrographs of oocytes (left panel) and embryos (right panel) at indicated stages. GV, germinal vesicle. MI, metaphase I. MII, metaphase II. Scale bars, 50 µm.

b. Schematic diagram showed the process from screening to successful delivery of a naturally aging monkey (T02).

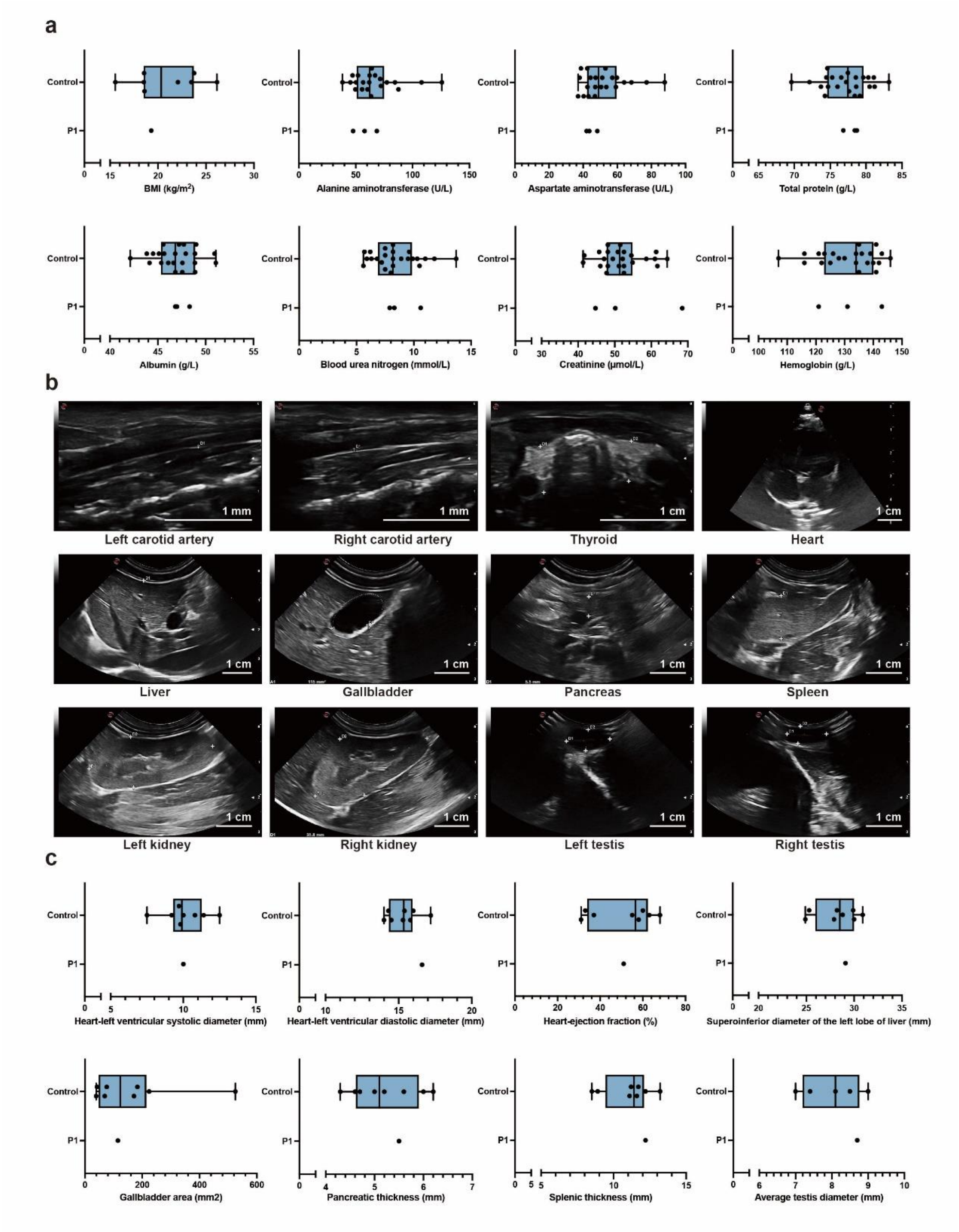


**Supplementary Fig. S7 The health and growth indicators of the offspring.**

a. Statistics of the BMI, serum levels of alanine aminotransferase (U/L), aspartate aminotransferase (U/L), total protein (g/L), albumin (g/L), blood urea nitrogen (mmol/L) and creatinine (μmol/L), and hemoglobin (g/L) in the offspring (P1) and the control cynomolgus monkeys.

b. Ultrasound observation of the bilateral carotid arteries, thyroid, heart, liver, gallbladder, pancreas, spleen, bilateral kidneys, and bilateral testes of the offspring

(P1). Scale bars as indicated.

c. Statistics of the Heart-left ventricular systolic diameter (mm), heart-left ventricular diastolic diameter (mm), heart-ejection fraction (%), super inferior diameter of the left lobe of liver (mm), gall-bladder wall thickness (mm), pancreatic thickness (mm), splenic thickness (mm), and testis size (mm) under ultrasound examination in the offspring (P1) and the control cynomolgus monkeys.

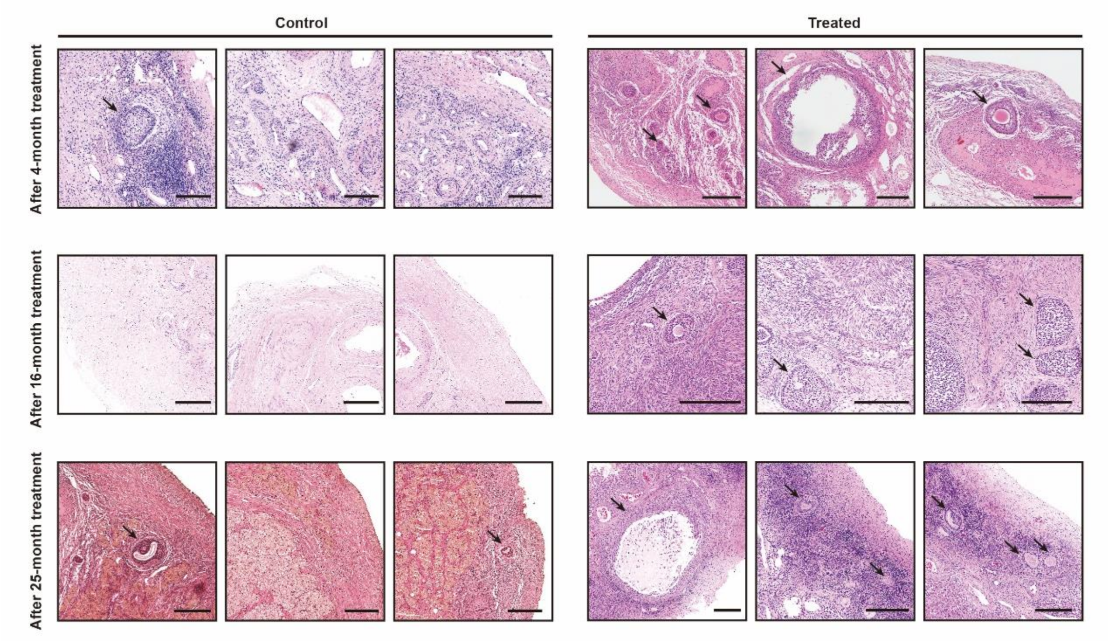


**Supplementary Fig. S8 M-cell transplantation promoted the follicle development of naturally aging monkeys.**

H&E-stained ovarian sections showed the growing follicles (black arrows) in the control and treated groups after 4-, 16-, and 25-month treatment, respectively. Scale bars, 200 μm.

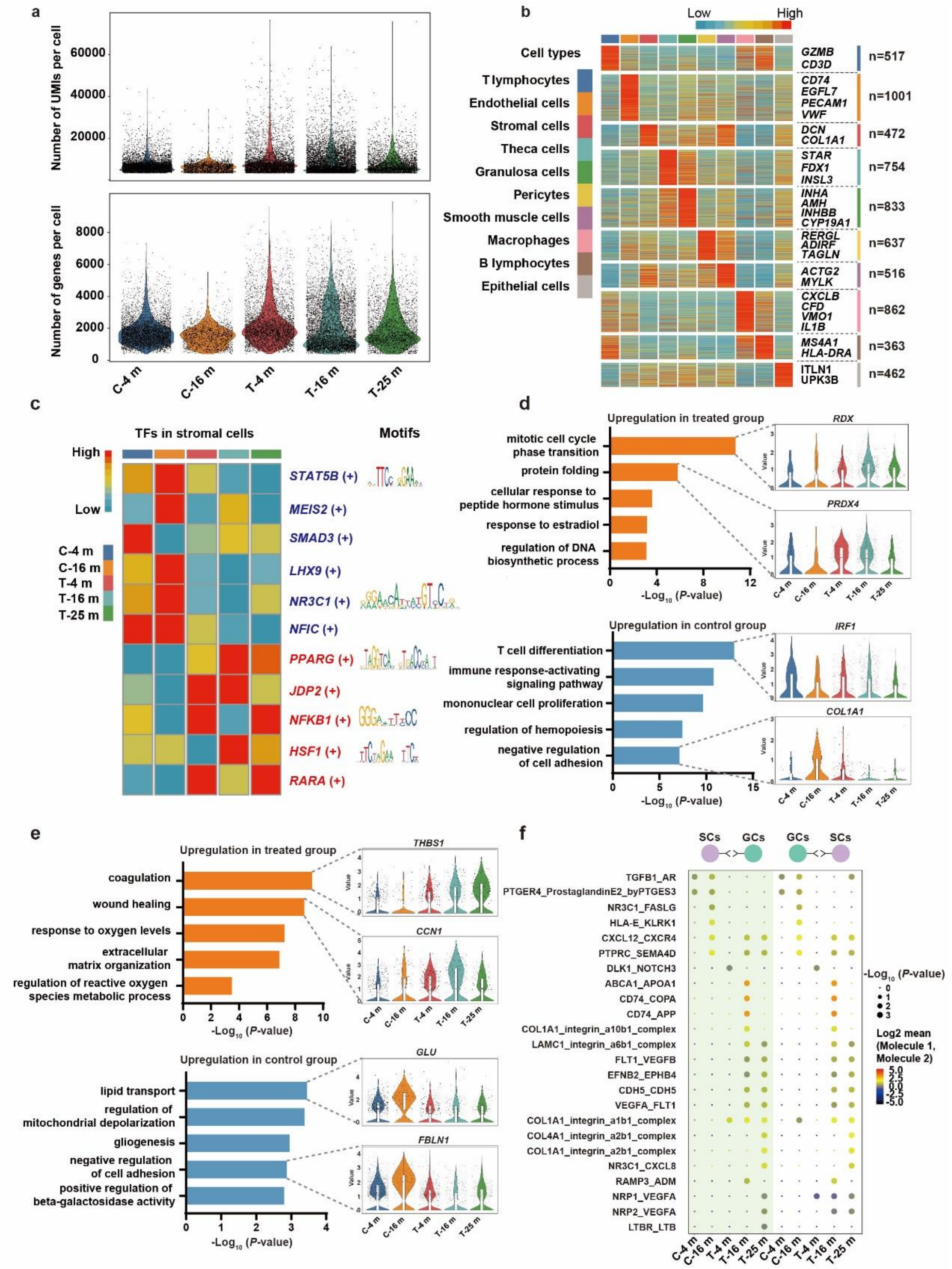


**Supplementary Fig. S9 A profile of transcription factors and target gene regulatory networks of GCs and SCs after treatment, as well as the cell bidirectional communication.**

a. Violin plots showed the number of detected unique molecular identifiers (UMIs, upper panel) and genes (bottom panel) in an individual cell.

b. Heatmap showed representative differentially expressed genes (DEGs) identified in

different cell populations. Gene expression levels were averaged and scaled. The color bar from blue to red indicated the relative expression levels from low to high.

**c**. Left panel: Heatmap showed the regulon activity score of representative transcriptional regulons in stromal cells of control and treated groups. Regulon activity scores were calculated by pySCENIC and were scaled for visualization. The color bar indicated the strength of regulon activity, with "High" (red) indicating active regulons and "Low" (blue) indicating inactive regulons. Right panel: Display of binding sites of some specific transcription factors.

d. Left panel: GO enrichment analysis of target genes of representative upregulated transcription factors in granulosa cells of the treated (upper) and control (bottom) groups. Right panel: Violin plots showed the expression levels of target genes related to the part of key GO terms.

e. Left panel: GO enrichment analysis of target genes of representative upregulated transcription factors in stromal cells of the treated (upper) and control (lower) groups. Right panel: Violin plots showed the expression levels of target genes related to the part of key GO terms.

f. Dot plot showed the representative ligand-receptor interactions of GC-SC. Interaction strength was measured using $log_2$-scaled means of the average expression level of the ligand in the indicated cell type and receptor in the other cell type. GCs, granulosa cells. SCs, stromal cells. Control group, C-4 m, and C-16 m. Treated group, T- 4 m, T-16 m, and T-25 m.

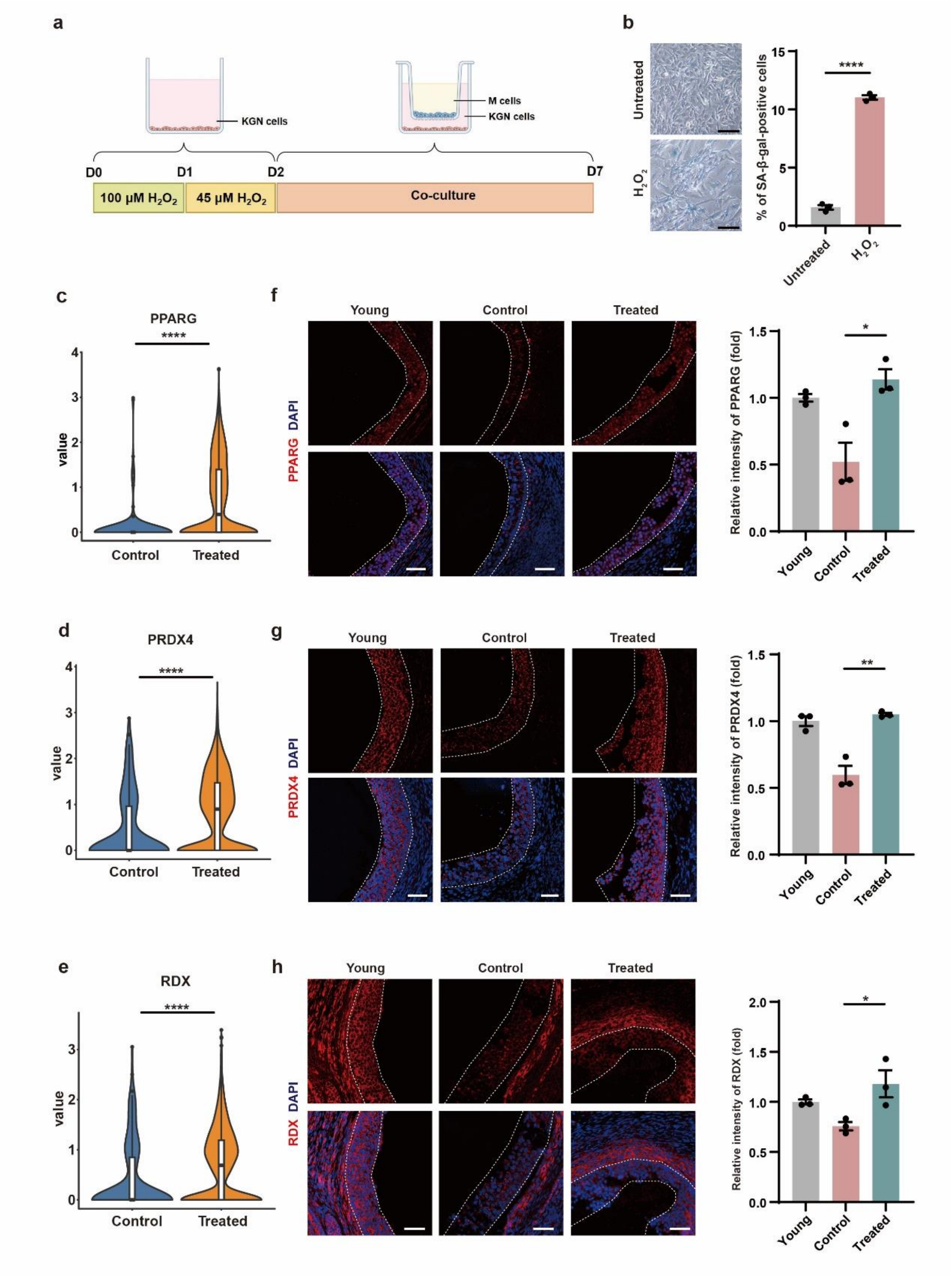


**Supplementary Fig. S10 Validation of the expression of *PPARG*, *PRDX4*, and *RDX* in ovaries.**

a. Schematic diagram showed the co-culturing of senescent KGN cells and M cells by Transwell culture system.

b. Senescence-associated beta-galactosidase (SA-β-gal) staining (left panel) on KGN cells with no treatment (untreated) and $H_2O_2$ treatment ($H_2O_2$). Scale bars, 100 μm. The proportion of SA-β-gal-positive cells after $H_2O_2$ treatment (right panel). n = 3,

****$P < 0.0001$ (two-tailed t-test).

c-e. Violin plots showed expression levels of *PPARG* (c), *PRDX4* (d), and *RDX* (e) in the GCs of control and treated groups by scRNA-seq analysis. n (control) = 2 monkeys, n (treated) = 3 monkeys, ****$P < 0.0001$ (two-tailed t-test).

f. Immunofluorescence analysis (left panel) showed the expression of PPARG in GCs of the young (3 y), control, and treated groups. Broken lines show boundaries of GCs in antral follicles. DAPI, DNA (here and after). Scale bars, 50 μm. Bar graph (right panel) illustrated the relative intensity of PPARG in GCs of young, control, and treated groups, respectively. n = 3, *$P < 0.05$ (two-tailed t-test).

g. Immunofluorescence analysis (left panel) showed the expression of PRDX4 in GCs of the young (3 y), control, and treated groups. Broken lines showed boundaries of GCs in antral follicles. Scale bars, 50 μm. Bar graph (right panel) illustrated the relative intensity of PRDX4 in GCs of young, control, and treated groups, respectively. n = 3, **$P < 0.01$ (two-tailed t-test).

h. Immunofluorescence analysis (left panel) showed the expression of RDX in GCs of the young, control, and treated groups. Broken lines showed boundaries of GCs in antral follicles. Scale bars, 50 μm. Bar graph (right panel) illustrated the relative intensity of RDX in GCs of young, control, and treated groups, respectively. n = 3, *$P < 0.05$ (two-tailed t-test).

.

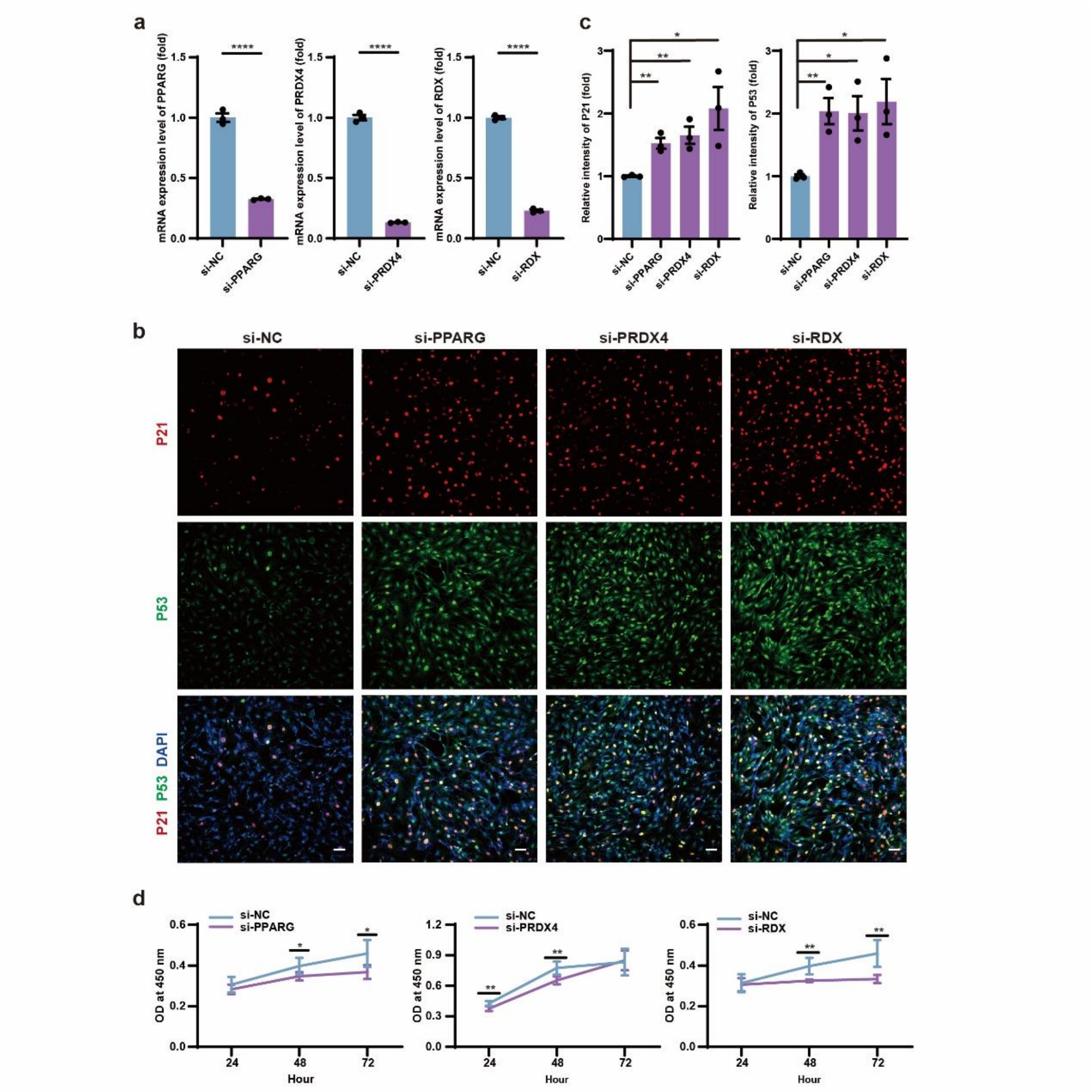


**Supplementary Fig. S11 *In vitro* verification of the molecular mechanisms of M-cell transplantation for alleviating ovarian aging.**

a. RT-qPCR showed the knockdown efficiency of KGN cells after transfection with negative control (si-NC) duplex or siRNAs against *PPARG* (left panel), *PRDX4* (middle panel), and *RDX* (right panel). n = 3, *****P* < 0.0001 (two-tailed t-test).

b. Immunofluorescent staining of P21 (red) and P53 (green) in KGN cells after *PPARG*-, *PRDX4*-, and *RDX*-knockdown. Scale bars, 50 μm.

c. Bar graphs showed the relative intensity of P21 (left panel) and P53 (right panel) in KGN cells after *PPARG*-, *PRDX4*-, and *RDX*-knockdown. n = 3, **P* < 0.05, ***P* < 0.01 (two-tailed t-test).

d. CCK8 assay of *PPARG*- (left panel), *PRDX4*- (middle panel), and *RDX*-knockdown (right panel) KGN cells. n = 6, **P* < 0.05 and ***P* < 0.01 (multiple t-tests).

# Supplementary Tables

**Supplementary Table S1. Information of the donors**

| Case no. | Indication for surgery | Age (y) | Age of Menarche (y) | Menstrual bleeding/ menstrual cycle | Number of pregnancies | Number of live births |
|---|---|---|---|---|---|---|
| 1 | Uterine adenosarcoma | 35 | 14 | 6/28-30 days | 0 | 0 |
| 2 | Cervical cancer | 36 | 15 | 5-7/30 days | 4 | 4 |
| 3 | Cervical malignancy | 36 | 16 | 4-5/38 days | 2 | 2 |
| 4 | Adenocarcinoma of the uterine Cervix | 37 | 13 | 5/26 days | 2 | 1 |
| 5 | Endometrial cancer | 38 | 15 | 7-10/27-28 days | 3 | 2 |
| 6 | Cervical malignancy | 42 | 18 | 3-4/23-24 days | 3 | 2 |
| 7 | Uterine endometrioid carcinoma | 42 | 13 | 7/30 days | 3 | 1 |
| 8 | Uterine endometrioid carcinoma | 42 | 13 | 10-15/27 days | 0 | 0 |
| 9 | Endometrial stromal sarcoma | 43 | 14 | 5-6/28-32 days | 1 | 1 |
| 10 | Cervical cancer | 44 | 13 | 4-6/28 days | 2 | 1 |
| 11 | Squamous cell carcinoma of the cervix | 45 | 13 | 5/30 days | 3 | 2 |
| 12 | Endometrial polyp | 45 | 14 | 3-7/30 days | 5 | 2 |
| 13 | Cervical malignancy | 45 | 12 | 3/28 days | 4 | 3 |
| 14 | Uterine endometrioid carcinoma | 46 | 13 | 4-5/30 days | 5 | 2 |
| 15 | Cervical intraepithelial neoplasia | 46 | 12 | 7/30 days | 5 | 3 |
| 16 | Cervical cancer | 47 | 16 | 2/28 days | 1 | 1 |
| 17 | Cervical high-grade squamous intraepithelial lesions | 47 | 13 | 4/30 days | 4 | 2 |
| 18 | Cervical adenosarcoma | 47 | 14 | 10/24 days | 2 | 1 |
| 19 | Uterine endometrioid carcinoma | 48 | 13 | 5/30 days | 3 | 2 |
| 20 | Uterine endometrioid carcinoma | 49 | 12 | 5-6/25 days | 2 | 2 |
| 21 | Atypical endometrial hyperplasia; Uterine leiomyoma; Uterine adenomyosis | 49 | 14 | 7-8/24-30 days | 3 | 1 |

| | | | | | | |
|---|---|---|---|---|---|---|
| 22 | Uterine leiomyoma; Uterine adenomyosis | 50 | 17 | 3/30+ days | 2 | 1 |
| 23 | Endometrioid adenocarcinoma | 50 | 14 | Menopause | 6 | 1 |
| 24 | Endometrial polyp; Uterine leiomyoma; Uterine adenomyosis | 51 | 12 | 7/28-30 days | 2 | 1 |
| 25 | Endometrial malignancy | 51 | 14 | 3/20 days | 4 | 2 |
| 26 | Uterine endometrioid carcinoma | 52 | 16 | Menopause | 1 | 1 |

**Supplementary Table S2. The number of primordial and primary follicles per ovary in humans**

| Case no. | Age (y) | Number of primordial follicles per ovary | Number of primary follicles per ovary |
|---|---|---|---|
| 1 | 35L | 4931 | 320 |
| 2 | 36R | 19979 | 2358 |
| 3 | 36R | 15987 | 1240 |
| 4 | 37L | 1800 | 71 |
| 5 | 38R | 12793 | 901 |
| 6 | 42L | 8873 | 474 |
| 7 | 42L | 13566 | 1233 |
| 8 | 42R | 1105 | 43 |
| 9 | 43R | 2574 | 889 |
| 10 | 44R | 7522 | 1043 |
| 11 | 45L | 2942 | 439 |
| 12 | 45R | 960 | 240 |
| 13 | 45L | 541 | 66 |
| 14 | 46L | 1850 | 71 |
| 15 | 46R | 418 | 46 |
| 16 | 47L | 1660 | 40 |
| 17 | 47L | 3422 | 826 |
| 18 | 47L | 177 | 35 |
| 19 | 48L | 57 | 0 |
| 20 | 49L | 74 | 37 |
|  | 49R | 77 | 77 |
| 21 | 49R | 54 | 54 |
| 22 | 50L | 280 | 112 |
|  | 50R | 305 | 34 |
| 23 | 50R | 0 | 0 |
| 24 | 51R | 172 | 86 |
| 25 | 51L | 147 | 0 |
| 26 | 52R | 0 | 0 |

L, left ovary; R, right ovary

**Supplementary Table S3. Ovarian follicle counts stratified by age group**

| Age group (y) | n | Average age (mean ± SEM) (y) | Number of primordial follicles per ovary (mean ± SEM) | Number of primary follicles per ovary (mean ± SEM) |
|---|---|---|---|---|
| 35-39 | 5 | 36.4 ± 0.5 | 11098.0 ± 3392.0 | 978.0 ± 402.2 |
| 40-44 | 5 | 42.6 ± 0.4 | 6728.0 ± 2246.0 | 736.4 ± 213.7 |
| 45-49 | 11 | 46.7 ± 0.5 | 1105.0 ± 363.6 | 170.4 ± 75.91 |
| 50-52 | 5 | 50.8 ± 0.4 | 122.4 ± 55.7 | 31.8 ± 19.58 |

**Supplementary Table S4. Physiological status of cynomolgus monkeys when first receiving injection**

| Animal No. | Age (y) | Weight (kg) | E2 (pg/mL) | P4 (ng/mL) | Injection |
|---|---|---|---|---|---|
| C01 | 23 | 3.5 | 27.27 | 0.08 | Saline |
| C02 | 20 | 5.4 | 58.73 | 0.08 | Saline |
| C03 | 20 | 5.2 | 86.60 | 0.33 | Saline |
| T01 | 20 | 5.1 | 74.98 | 0.22 | M cells |
| T02 | 20 | 3.4 | 60.6 | 0.30 | M cells |
| T03 | 20 | 4.0 | 69.15 | 0.47 | M cells |
| T04 | 20 | 4.5 | 84.52 | 0.22 | M cells |
| T05 | 19 | 5.2 | 84.28 | 0.27 | M cells |
| T06 | 19 | 4.3 | 97.16 | 0.12 | M cells |
| T07 | 18 | 5.5 | 43.75 | 0.13 | M cells |

**Supplementary Table S5 Experimental design for each monkey.**

| Animal No. | Hormone measurement | Naturally mating | Unilateral oophorectomy | Super-ovulation | Histological analysis | scRNA-seq |
|---|---|---|---|---|---|---|
| **C01** | -2, 3, 6, 8 months | 2 months | 4 months | - | 4 months | 4 months |
| **C02** | -2, 3, 6, 8 months | 2 months | - | 5 months | 25 months | - |
| **C03** | -2, 3, 6, 8 months | 2 months | - | 5 months | 16 months | 16 months |
| **T01** | -2, 3, 6, 8 months | 2 months | 4 months | - | - | 4 months |
| **T02** | -2, 3, 8 months | 2 months (pregnancy) | - | - | 25 months | - |
| **T03** | -2, 3, 6, 8 months | 2 months | - | 5 months | - | 25 months |
| **T04** | -2, 3, 6, 8 months | 2 months | - | 5 months | 16 months | 16 months |
| **T05** | -2, 3, 6, 8 months | 2 months | - | 5 months | - | - |
| **T06** | -2, 3, 6, 8 months | 2 months | 4 months | 5 months | 4 months | - |
| **T07** | -2, 3, 6 months | 2 months | - | - | - | - |

-2 months, before 2-month treatment. 2, 3, 4, 5, 6, 8, 16, 25 months, after 2-, 3-, 4-, 5-, 6-, 8-, 16-, 25-month treatment.

**Supplementary Table S6. Primer sequences used for RT-qPCR**

| Name | Sequences |
|---|---|
| Human-GAPDH-F (5’-3’) | TCGGAGTCAACGGATTTGGT |
| Human-GAPDH-R (5’-3’) | TTGCCATGGGTGGAATCATA |
| Human-Radixin-F (5’-3’) | GAATTTGCCATTCAGCCCAATA |
| Human-Radixin-R (5’-3’) | GCCATGTAGAATAACCTTTGCTGTC |
| Human-PPARG-F (5’-3’) | GGGATCAGCTCCGTGGATCT |
| Human-PPARG-R (5’-3’) | TGCACTTTGGTACTCTTGAAGTT |
| Human-PRDX4-F (5’-3’) | AGAGGAGTGCCACTTCTACG |
| Human-PRDX4-R (5’-3’) | GGAAATCTTCGCTTTGCTTAGGT |
| Human-P16-F (5’-3’) | CTCGTGCTGATGCTACTGAGGA |
| Human-P16-R (5’-3’) | GGTCGGCGCAGTTGGGCTCC |
| Human-P21-F (5’-3’) | CTCGTGCTGATGCTACTGAGGA |
| Human-P21-R (5’-3’) | GGTCGGCGCAGTTGGGCTCC |
| Human-P53-F (5’-3’) | CCTCAGCATCTTATCCGAGTGG |
| Human-P53-R (5’-3’) | TGGATGGTGGTACAGTCAGAGC |
| Human-IL1A-F (5’-3’) | TGTAAGCTATGGCCCACTCCA |
| Human-IL1A-R (5’-3’) | AGAGACACAGATTGATCCATGCA |
| Human-IL6-F (5’-3’) | ACTCACCTCTTCAGAACGAATTG |
| Human-IL6-R (5’-3’) | CCATCTTTGGAAGGTTCAGGTTG |
